# Dual communities in spatial networks

**DOI:** 10.1038/s41467-022-34939-6

**Published:** 2022-12-03

**Authors:** Franz Kaiser, Philipp C. Böttcher, Henrik Ronellenfitsch, Vito Latora, Dirk Witthaut

**Affiliations:** 1 Forschungszentrum Jülich, Institute for Energy and Climate Research (IEK-STE), 52428 Jülich, Germany; 2 Institute for Theoretical Physics, University of Cologne, 50937 Köln, Germany; 3 Physics Department, Williams College, 33 Lab Campus Drive, Williamstown, MA 01267 USA; 4 Department of Mathematics, Massachusetts Institute of Technology, Cambridge, MA 02139 USA; 5 School of Mathematical Sciences, Queen Mary University of London, London, E1 4NS UK; 6 Dipartimento di Fisica ed Astronomia, Università di Catania and INFN, 95123 Catania, Italy; 7 Complexity Science Hub Vienna, 1080 Vienna, Austria

**Keywords:** Applied mathematics, Energy grids and networks, Complex networks, Biological physics

## Abstract

Both human-made and natural supply systems, such as power grids and leaf venation networks, are built to operate reliably under changing external conditions. Many of these spatial networks exhibit community structures. Here, we show that a relatively strong connectivity between the parts of a network can be used to define a different class of communities: dual communities. We demonstrate that traditional and dual communities emerge naturally as two different phases of optimized network structures that are shaped by fluctuations and that they both suppress failure spreading, which underlines their importance in understanding the shape of real-world supply networks.

## Abstract (web summary)

Here the authors introduce dual communities, characterized by strong connections at their boundaries, and show that they are formed as a trade-off between efficiency and resilience in supply networks.

## Introduction

Community structures are a fundamental trait of complex networks and have found numerous applications in systems ranging from social networks^1^ to biological networks^2,3^ and critical infrastructures such as power grids^4^. Typically, communities are defined by a strong connectivity within the community compared to a relatively weak connectivity between different communities^1,5,6^. They may correspond to functional units of the network, for instance in metabolic networks^7^, or actual communities in social networks.

Intuitively, community structures play an important role for the spreading of failures or perturbations in networked systems. The low connectivity impedes spreading processes, such that perturbations can be expected to stay within the community, which is both predicted by theory^8,9^ as well as observed in experiments^10^. Community structures thus provide robustness in complex networks, but other structural patterns may have a comparable effect. In particular, it has been shown that hierarchical structures may provide similar features, for instance in vascular networks of plants^11,12^.

In this article, we provide a unified view on the role of communities and hierarchies for network robustness based on the concept of graph duality. The dual graph is most naturally defined for spatially embedded networks, i.e., networks that are embedded in the plane without edges crossing each other. This class of networks includes a large variety of man-made and biological systems^13–16^. The vertices of the dual correspond to the faces of the original, primal graph. Two dual vertices (faces) are connected if they share at least one edge. Graph duality has been previously used to study fixed points in oscillator networks^17,18^ and to speed up network algorithms^19,20^. Here we use this concept to reveal patterns in the network structure that are hidden in the primal graph. In particular, we establish dual communities—communities that are defined within the dual graph—and highlight their relation to hierarchical network structures. Furthermore, graph duality readily explains why both weak and strong connections can make a network robust: strong connections in the primal translate into weak connections in the dual and vice versa.

The article is organized as follows. We first demonstrate how different structural patterns impede spreading processes and thus contribute to the robustness of a network. Focusing on flow networks, we formalize the concept of graph duality and establish dual communities. Second, we investigate essential properties of dual communities, in particular their link to hierarchical patterns and the geometry of the community boundaries. Finally, we study the emergence and impacts of communities structures. Using optimization models, we study why networks develop a primal or a dual community structure. We provide a deeper analysis of the link between communities and network robustness by employing different simulation models. Throughout the article we use the terms graph and network interchangeably. The term “graph” stresses the structural aspects while “network” stresses the functional aspects of the system.

## Results

### Network robustness and community structure

**Fig. 1:**
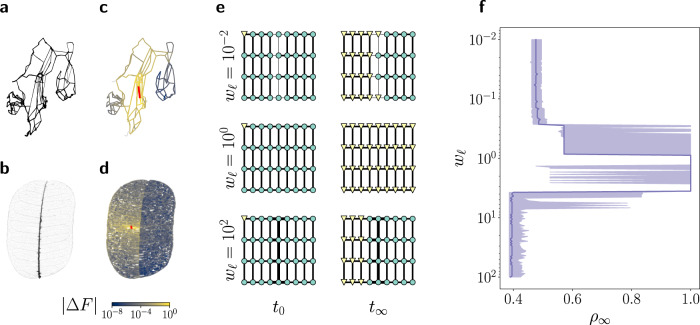
Different structural patterns separate networks and increase network robustness. **a** Topology of the Scandinavian power grid, with weak connectivity between different geographic units, in particular Finland. **b** Venation network of the leaf *Schizolobium amazonicum*, with a strong central vein separating the leaf into left and right. The width of the lines in **a**, **b** encode the edge weights. **c**, **d** Flow changes ∣Δ*F*∣ after the failure of a single edge (colored in red) for the two networks shown in **a**, **b**. The impact of the failure is strongly suppressed in another part of the network, i.e., in Finland and the right half of the leaf, respectively. This highlights the existence and impact of boundaries that separate the network into communities. **e** Simulation of a classic model of global cascades^24,25^ in a lattice with inhomogenous edge weights. Edges in the middle have weight *w*_*ℓ*_ (indicated by thin/thick lines), while all other edges have a weight of *w*_*ij*_ = 1 (see “Methods” for details). Infected/faulty nodes are shown as yellow triangles, healthy/operational nodes as green circles. A global cascade occurs for a homogeneous lattice (*w*_*ℓ*_ = 1), while both weak (*w*_*ℓ*_ = 10^−2^) and strong connections (*w*_*ℓ*_ = 10^2^) stop the cascade from propagating to the right part of the network. **f** Final fraction *ρ*_*∞*_ of nodes that become infected/faulty during the cascade as a function of the weight parameter *w*. The line represents the median and the shaded region the 25–75% quantile for 1000 random initial conditions. For homogeneous lattices (*w* = 1), the cascade reaches all nodes (*ρ*_*∞*_ = 1). For both weak (*w* ≪ 1) and strong (*w* ≫ 1) conditions, the cascade stops at the boundary such that *ρ*_*∞*_ ≈ 0.5.

We first highlight how network robustness is related to the presence of communities for selected examples. Robustness is essential for critical infrastructures such as electric power grids. The high-voltage transmission grid of Scandinavia (Fig. 1a, c) has an obvious community structure due to geographic reasons. Finland is only weakly connected to the rest of Scandinavia through two high-voltage transmission lines. We simulate the impact of a transmission line failure, which is the biggest threat for large-scale blackouts^21^. We use the linear power flow or DC approximation^22,23^, which will be described in detail below. The flow changes after the failure generally decay with the distance to the failing transmission line, but we also observe a strong impact of the community boundary. Flow changes are strongest in the community where the failure occurs, in this case Sweden. They are substantially suppressed in the other community, i.e., Finland, which also reduces the risk of a global cascade of failures.

Remarkably, a similar suppression of failure spreading is realized by strong connections. We consider the venation network of a leaf, which includes a strong central vein separating the left and right half (Fig. 1b, d). The flow of water and nutrients is described by a linear flow network^11^, which is mathematically equivalent to the linear power flow approximation. If a secondary vein fails, we observe a very similar picture as for the power grid: flow changes generally decay and are strongly suppressed in the other half of the leaf. The central vein itself features large flow changes and thus provides a buffer function. We conclude that weak and strong connections can equally suppress the spreading of failures. We will show that both effects are fully equivalent and that they can be understood in terms of network communities, provided we generalize the definition of communities.

Before we move to a more detailed analysis, we demonstrate the generality of the observed phenomena. We consider a classic model of network cascades^24,25^. Nodes are either healthy/operational (state 0) or infected/faulty (state 1). A node *i* gets infected or faulty if the weighted average over all neighbors’ states exceeds a certain threshold *ϕ*_*i*_. Starting from a small amount of nodes in state 1, a cascade may emerge depending on the values *ϕ*_*i*_ and the structure of the network. As before, we consider networks which can be separated into two parts by either weak or strong connections (Fig. 1e, f). More precisely, we consider a lattice where the edges in the middle region have a tunable weight *w*, while all other edges have weight one (see “Methods” for details). We observe that a homogeneous network (*w* = 1) always leads to a global cascade, where all nodes are infected or faulty in the final state. A boundary, either by weak (*w* ≪ 1) or strong (*w* ≫ 1) connections, effectively stops the cascade. Only those nodes are infected, which are located in the same half as the initially infected ones.

### Flow networks and dual communities

We have shown that strong connections can divide a network and enhance its robustness similarly as weak connections do. Even more, we can establish a full mathematical equivalence of weak and strong connections in the case of flow networks. This equivalence leads to a generalization of the definition of community structures in complex networks.

Linear flow networks arise in a variety of applications, including electric circuits^26,27^, power grids^22,23^, hydraulic networks^28,29^, and vascular networks of plants^11^. In these networks, the flow from node *i* to node *j* is given by *F*_*i*→*j*_ = *w*_*ij*_ ⋅ (*θ*_*i*_ − *θ*_*j*_), where *w*_*ij*_ is the connectivity or conductivity of the edge (*i*, *j*). The nodal variable *θ*_*i*_ describes the local voltage or potential in an electric circuit, the voltage phase angle in a power grid, or the pressure in a hydraulic or vascular network. The flows have to satisfy the continuity equation (or Kirchhoff’s current law, KCL) at every node *i* of the network, ∑_*j*_* F*_*i*→*j*_ = *P*_*i*_, where *P*_*i*_ is the inflow to the network.

These equations can be recast in a compact matrix notation. Let *N* denote the number of nodes and *M* the number of edges in the network, which we assume to be connected. We fix an orientation for each edge to keep track of the direction of flows and define the edge-node incidence matrix ${{{{{{{\bf{I}}}}}}}}\in {{\mathbb{R}}}^{M\times N}$ as

1 $${I}_{\ell n}=\left\{\begin{array}{ll}+1&{{{{{{{\rm{if}}}}}}}}\,{{{{{{{\rm{edge}}}}}}}}\,\ell \,{{{{{{{\rm{starts}}}}}}}}\,{{{{{{{\rm{at}}}}}}}}\,{{{{{{{\rm{node}}}}}}}}\,n,\\ -1&\!{{{{{{{\rm{if}}}}}}}}\,{{{{{{{\rm{edge}}}}}}}}\,\ell \,{{{{{{{\rm{ends}}}}}}}}\,{{{{{{{\rm{at}}}}}}}}\,{{{{{{{\rm{node}}}}}}}}\,n,\\ 0&{{{{{{{\rm{otherwise}}}}}}}}.\hfill\end{array}\right.$$

The edge weights are summarized in a diagonal matrix **W** = diag(*w*_1_, …, *w*_*M*_) while all other quantities are summarized in vectors ${{{{{{{\boldsymbol{\theta }}}}}}}}={({\theta }_{1},\ldots,\, {\theta }_{N})}^{\top }$, ${{{{{{{\bf{P}}}}}}}}={({P}_{1},\ldots,\, {P}_{N})}^{\top }$, ${{{{{{{\bf{F}}}}}}}}={({F}_{1},\ldots,\, {F}_{M})}^{\top }$. Note, the ordering of the edges in the edge-node incidence matrix **I** and the weights in the diagonal matrix **W** have to be consistent such that the weight of edge *k* connecting nodes *i* and *j* is given by *w*_*k*_ = *w*_*ij*_. Then the relation of flows and potentials is given by **F** = **WI*θ*** and Kirchhoff’s current law reads

2 $${{{{{{{\bf{P}}}}}}}}={{{{{{{{\bf{I}}}}}}}}}^{\top }{{{{{{{\bf{F}}}}}}}}=\underbrace{{{{{{{{{\bf{I}}}}}}}}^{\top }{{{{{{{\bf{W}}}}}}}}{{{{{{{\bf{I}}}}}}}}}}_{\begin{array}{c}=\!\!\!:{{{{{{{\bf{L}}}}}}}}\end{array}}\,{{{{{{{\boldsymbol{\theta }}}}}}}}.$$

Equation (2) is a discrete Poisson equation that determines the potential ***θ*** up to an irrelevant additive constant. The matrix ${{{{{{{\bf{L}}}}}}}}\in {{\mathbb{R}}}^{N\times N}$ is nothing but the well known graph Laplacian with components

3 $${L}_{ij}=\left\{\begin{array}{ll}-{w}_{ij}&{{{{{{{\rm{if}}}}}}}}\,i\,{{{{{{{\rm{is}}}}}}}}\,{{{{{{{\rm{connected}}}}}}}}\,{{{{{{{\rm{to}}}}}}}}\,j,\\ {\sum }_{k}{w}_{ik}&{{{{{{{\rm{if}}}}}}}}\,i=j,\hfill\\ 0&{{{{{{{\rm{otherwise}}}}}}}}.\hfill\end{array}\right.$$

The Laplacian is a central object in spectral graph bisection^30^, a classic method of community detection, which will be further elaborated below.

The above description focuses on the nodes of the network, with the nodal potentials ***θ*** being the central quantity of interest. An equivalent description exists that focuses on the edges of the network and the flows **F**. The starting point is the KCL **I**^⊤^**F** = **P**. This linear set of equations is underdetermined in terms of **F**, such that the general solution can be written as the sum of a particular solution and an arbitrary solution of the associated homogeneous equation, namely

4 $${{{{{{{\bf{F}}}}}}}}={{{{{{{{\bf{F}}}}}}}}}_{{{{{{{{\rm{part}}}}}}}}}+{{{{{{{{\bf{F}}}}}}}}}_{\hom }.$$

The vector **F**_hom_ describes a flow without sources or sinks, that is, a collection of cycle flows. The cycle flows form a vector space (the cycle space) such that we can expand each cycle flow into a suitable basis. A distinguished basis exists for plane graphs, i.e., graphs embedded in the plane, which can be constructed in the following way. A face of a plane graph is a region that is bounded by edges, but contains no edges in the interior. The boundary edges of each face then provide one basis vector of the cycle space. Further details are given in the Supplementary Information.

To keep track of the basis, we introduce the cycle edge incidence matrix ${{{{{{{\bf{C}}}}}}}}\in {{\mathbb{R}}}^{M\times (M-N+1)}$ with components

5 $${C}_{\ell c}=\left\{\begin{array}{ll}+1&{{{{{{{\rm{if}}}}}}}}\,{{{{{{{\rm{edge}}}}}}}}\,\ell \,{{{{{{{\rm{is}}}}}}}}\,{{{{{{{\rm{part}}}}}}}}\,{{{{{{{\rm{of}}}}}}}}\,{{{{{{{\rm{face}}}}}}}}\,c,\hfill\\ -1&{{{{{{{\rm{if}}}}}}}}\,{{{{{{{\rm{reversed}}}}}}}}\,{{{{{{{\rm{edge}}}}}}}}\,\ell \,{{{{{{{\rm{is}}}}}}}}\,{{{{{{{\rm{part}}}}}}}}\,{{{{{{{\rm{of}}}}}}}}\,{{{{{{{\rm{face}}}}}}}}\,c,\\ 0&{{{{{{{\rm{otherwise}}}}}}}}.\hfill\end{array}\right.$$

Then we can write the general solution of the KCL as

6 $${{{{{{{\bf{F}}}}}}}}={{{{{{{{\bf{F}}}}}}}}}_{{{{{{{{\rm{part}}}}}}}}}+{{{{{{{\bf{C}}}}}}}}\,{{{{{{{\bf{f}}}}}}}}$$

with an arbitrary cycle flow vector **f**. The actual values of the cycle flows are then determined by Kirchhoff’s voltage law (KVL), which states that the potential differences around any closed cycle sum up to zero. In fact it is sufficient to enforce this for the *M* − *N* + 1 basis cycles. We can thus formulate the KVL in terms of the flow vector **F** as

7 $${{{{{{{{\bf{C}}}}}}}}}^{\top }{{{{{{{{\bf{W}}}}}}}}}^{-1}{{{{{{{\bf{F}}}}}}}}={{{{{{{\bf{0}}}}}}}}.$$

Crucially, this equation includes the matrix **W**^−1^ which translates flows into potential differences. Substituting Eq. (6) then yields

8 $${{{{{{{\bf{Q}}}}}}}}=\underbrace{{{{{{{{{\bf{C}}}}}}}}^{\top }{{{{{{{{\bf{W}}}}}}}}}^{-1}{{{{{{{\bf{C}}}}}}}}}}_{\begin{array}{c}={{{{{{{{\bf{L}}}}}}}}}^{*}\end{array}}\,{{{{{{{\bf{f}}}}}}}}.$$

Notably, this equation has the same mathematical structure as Eq. (2): It is a discrete Poisson equation with a Laplacian matrix L^*^ and a source term **Q** = −**C**^⊤^**W**^−1^**F**_part_. However, the Laplacian L^*^ is not defined on the original primal graph, but on the dual graph. The vertices of this dual graph are given by the faces of the primal graph, while two nodes of the dual graph are connected by a dual edge if the corresponding faces share an edge.

Comparing the Laplacian of the primal graph **L** = **I**^⊤^**WI** to that of the dual graph L^*^ = **C**^⊤^**W**^−1^**C**, we see another essential aspect of graph duality: The weights of the dual edges are inverse to the weights of the primal edges. More precisely, we find the dual weights

9 $${w}_{c,d}^{*}=\mathop{\sum }_{\ell \in c,d}\frac{1}{{w}_{\ell }}$$

of the edge that connects the nodes *c* and *d* in the dual graph corresponding to faces *c* and *d* that share the edge *ℓ* in the primal graph. This relation shows most clearly why weak and strong connections can both affect the robustness and the community structure of a network. Strong connections in the primal correspond to weak connections in the dual and vice versa. Similarly, a strong central vein in the primal corresponds to weak connections in the dual and thus to a pronounced community structure.

We have now introduced all mathematical tools to identify dual communities in planar complex networks. Starting from the primal network, we identify all faces and define the dual graph with weights given by Eq. (9). Then dual communities can be extracted by means of any standard community detection algorithm.

In the following, we focus on spectral graph bisection because of its direct link to the graph Laplacian—which is the central object in the above analysis. This method relies on the fact that the community structure is encoded in the second smallest eigenvalue of the graph Laplacian *λ*_2_ ≥ 0, known as the algebraic connectivity or Fiedler value, which vanishes if the graph consists of two disconnected components and increases with increasing connectivity between the communities. The graph nodes are then assigned to one of two communities based on the corresponding eigenvector **v**_2_, the Fiedler vector: two vertices *j* and *i* are in the same community if they share the same sign of the Fiedler vector^5^.

10 $${{{{{{\mathrm{sign}}}}}}}\,({({{{{{{{{\bf{v}}}}}}}}}_{2})}_{i}-h)={{{{{{\mathrm{sign}}}}}}}\,({({{{{{{{{\bf{v}}}}}}}}}_{2})}_{j}-h),$$

where $h\in {\mathbb{R}}$ is a threshold parameter. Here, we choose *h* = 0.

**Fig. 2:**
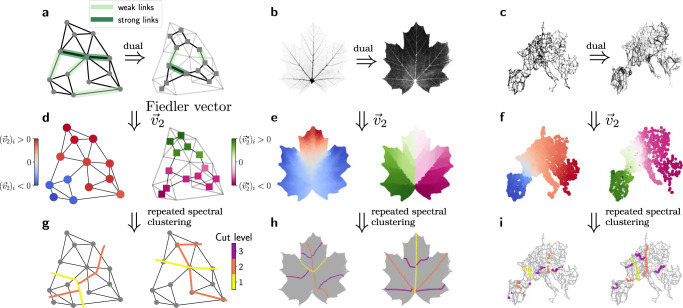
Primal and dual communities and hierarchies in spatial networks. **a** A plane graph with edges characterized by either large (dark green), small (light green) or intermediate edge weights, and its associated dual graph. The dual graph is constructed by transforming each face of the graph into a node of the dual, and adding dual edges whenever two faces share an edge. Notice that an edge with large weight shared by two faces will imply a weak link between the two corresponding nodes in the dual graph (see Eq. (9)). **d** Spectral clustering by means of the Fiedler vector **v**_2_ reveals the community structure in both the graph (left) and its dual (right). **g** Based on repeated spectral clustering, the graphs are further decomposed into a hierarchy of smaller sub-units which is different in the graph (left) and its (dual). **b**, **e**, **h** If we perform the same analysis on the venation network of a leaf of *Acer platanoides*, a decomposition of the original graph does not provide useful information (**e**, **h**, left). A decomposition of the dual graph, however, reveals the hierarchical organization and the functional units of the venation network (**e**, **h**, right). **c**, **f**, **i** Applying the same procedure to the Central European power grid, (**i**, left) and its dual (**i**,right) show that primal and dual hierarchies provide different, but equally useful information about the network structure. We may conclude that this network represents an intermediate case between primal and dual community structure (see main text for details).

This method can be straightforwardly applied to the dual graph, replacing the primal Laplacian **L** by its dual counterpart L^*^. The algebraic connectivity of the dual is measured by the second eigenvalue ${\lambda }_{2}^{*}$ and the associated eigenvector is used to identify the dual communities. We find that dual communities appear naturally in real-world networks such as the venation networks of leaves (Fig. 2b, e). In the following, we discuss essential properties of dual communities, in particular their relation to hierarchical structures, and provide a thorough analysis of the dual algebraic connectivity ${\lambda }_{2}^{*}$. We stress that other community detection methods can be applied to the dual graph equally well and yield comparable results (cf. Supplementary Fig. S6).

### Dual communities reveal hierarchical organization of supply networks

The spectral clustering method for community detection can be applied to both the primal and the dual graph, revealing different structural information about the network (Fig. 2). Furthermore, we can use this approach to extract a network’s hierarchical organization as follows. Starting from the initial network, we compute the Fiedler vector, identify the communities and then split the network into two parts at the resulting boundary by removing all edges between the communities. Then we iterate the procedure starting from the subgraphs obtained in the previous step. Repeated application of this procedure reveals different boundaries and thus different hierarchies in the primal graph and in its dual (Fig. 2g, see “Methods” for details).

Leaf venation networks are archetypal examples of hierarchically organized networks, with a thick primary vein in the middle and medium secondary veins that supply thin subordinated veins (Fig. 2b). The thick veins separate the network into distinct parts—for instance, the left and right half separated by the primary vein. This characteristic organization is clearly revealed by dual community detection. Spectral graph bisection identifies the primary vein that separates the left and the right half of the leaf. Repeating the bisection then shows that this organizational pattern repeats in a hierarchical order: dual communities are split by secondary veins in a repeated manner (Fig. 2h). Remarkably, an analog decomposition in the original primal graph does not provide any useful information on the network organization.

We conclude that leaf venation networks clearly display a dual community structure, where the boundary of the dual communities coincide with the primary and secondary veins. Hence, dual community detection allows to identify hierarchical organization patterns in complex networks. We will provide a more formal treatment of the relation between strong veins and dual communities below.

As a second example, we now turn to another type of spatially embedded supply networks: power grids. Figure 2c shows the European power transmission grid and its dual graph. Again, a hierarchical decomposition reveals different levels of hierarchies in the grid that correspond to its functional components. These components may also be interpreted geographically: the mountain ranges such as the Pyrenees or the Alps as well as the former Iron Curtain are clearly visible in the decomposition of the primal graph. Remarkably, both primal and dual decompositions provide useful structural information here. In particular, there is a dual community boundary at cut level three that spans Hungary and the border region between Slovenia and Croatia and closely corresponds to a weak spot in the European power system, where the grid was split into two mutually asynchronous fragments on January 8, 2021^31^. Another split occurred on the 24th of July between Spain and France—where both the primal and the dual decomposition detect a community boundary^32^.

Although mathematically similar^12,33,34^, the two types of networks we studied display different structural hierarchies and communities. Whereas leaf venation networks are evolutionarily optimized, the structure of power grids depends strongly on historical aspects and their ongoing transition to include a higher share of renewable energy sources. This transition aspect also manifests in their community structure, as we will see further below.

### Connectivity and the geometry of community boundaries

**Fig. 3:**
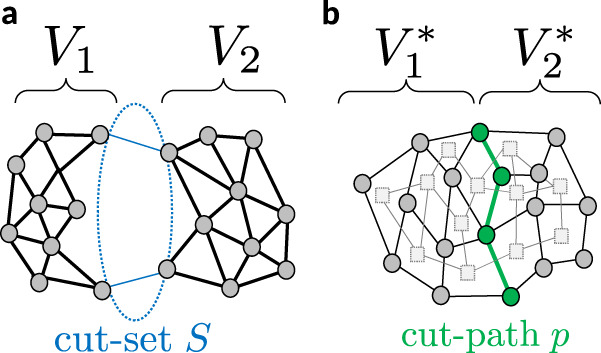
Geometry of primal and dual community boundaries. **a** Decomposition of a graph into two primal communities. Some edges belong to neither of the two communities, but provide a weak connection between the communities. These edges constitute the cut-set *S*. **b** A graph (black solid lines) and its dual (gray dashed lines), which is decomposed into two communities ${V}_{1,2}^{*}$. Some primal edges, colored in green, belong to faces from both communities. These primal edges, together with their terminal nodes, constitute the cut-path *p*.

The algebraic connectivity *λ*_2_ of a graph is closely related to its topological connectivity—the amount of connectivity between the two communities^35^. For a weighted graph, one can derive the upper bound (see Supplementary Note 2)

11 $${\lambda }_{2}\le {\mu }_{2}=\frac{{N}_{1}+{N}_{2}}{{N}_{1}{N}_{2}}\mathop{\sum }_{\ell \in S}{w}_{\ell },$$

where *N*_1_ and *N*_2_ respectively count the number of nodes in the two communities. The set *S* contains all edges which are not within one of the two communities but in between, providing a weak connection of the communities (Fig. 3a). This set is referred to as a cut-set: If all edges in *S* are removed, the graph is cut into the two communities. Notably, the bound becomes exact in the limit of vanishing connectivity (*μ*_2_ → 0) as shown in the Supplementary Information.

We derive an analogous bound for dual communities, transferring geometric concepts from the primal to the dual. In particular, we derive an analog to the cut-set *S*, which contains all edges, which are elements of neither of the two components. Consider a decomposition of the dual graph G^*^ = (V^*^, E^*^), where the dual vertex set V^*^ is separated into two components ${V}_{1}^{*}$ and ${V}_{2}^{*}$. Two faces $c\in {V}_{1}^{*}$ and $d\in {V}_{2}^{*}$ are connected in the dual, if they share at least one edge in the primal graph. Hence, we will find a set of primal edges which belong to both of the two components (Fig. 3b). These primal edges, together with their terminal vertices, constitutes a path *p* in the primal graph. In the following, we will refer to *p* as a cut-path as its removal disconnects the graph. The edges along the cut-path essentially determine the community structure of the dual graph and its algebraic connectivity. Given a cut-path *p*, we find the bound

12 $${\lambda }_{2}^{*}\le {\mu }_{2}^{*}=\frac{{N}_{1}^{*}+{N}_{2}^{*}}{{N}_{1}^{*}{N}_{2}^{*}}\mathop{\sum }_{\ell \in p}\frac{1}{{w}_{\ell }}\,,$$

where ${N}_{1,2}^{*}=|{V}_{1,2}^{*}|$ counts the number of nodes in the dual communities. Notably, the expression ${\mu }_{2}^{*}$ does not only provide an upper bound for the algebraic connectivity ${\lambda }_{2}^{*}$, but an approximation that becomes exact in the limit of vanishing dual connectivity. We prove these statements rigorously in the Supplementary Information.

**Fig. 4:**
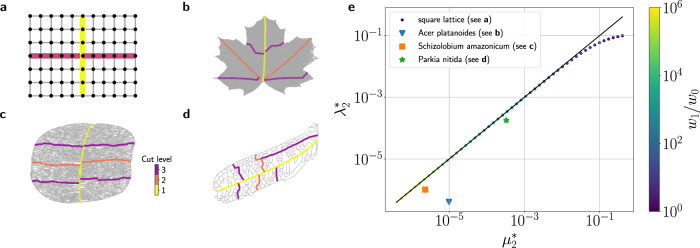
Algebraic and Topological Connectivity in the dual graph of synthetic and real-world networks. **a** A square lattice with a tunable dual community structure. The edge weights along the central vein *w*_1_ are stronger than the weights *w*_0_ of the remaining edges. The ratio *w*_1_/*w*_0_ can be tuned to test the relation of algebraic and topological connectivity given by Eq. (12). **b**–**d** Hierarchical organization in leaf venation networks of three different species revealed by repeated graph bisection of the dual graph. The dual community boundary (yellow) constitutes a cut-path *p*. **e** Comparison of the dual algebraic connectivity ${\lambda }_{2}^{*}$ and the dual topological connectivity ${\mu }_{2}^{*}$ defined in Eq. (12). Circles refer to the case of the square lattice with the color of the circles indicating the fraction *w*_1_/*w*_0_. The topological connectivity ${\mu }_{2}^{*}$ provides a rigorous upper bound for ${\lambda }_{2}^{*}$, but also a good approximation for large parameter regions. This correspondence shows how dual communities are decomposed by a strong connectivity along the boundary.

The relation of cut-paths and dual communities is further investigated in Fig. 4 for both synthetic networks and leaf venation networks. We first consider a square lattice with a tunable dual community structure: The edges in the central vein have a higher weight *w*_1_ than the remaining edges *w*_0_. We find that the dual algebraic and topological connectivity ${\lambda }_{2}^{*}$ and ${\mu }_{2}^{*}$ become virtually indistinguishable for *w*_1_/*w*_0_ ≳ 10^2^. In venation networks, the boundaries between the dual communities, i.e., the cut-paths, correspond to the primal and secondary veins as described above. A good agreement between ${\mu }_{2}^{*}$ and ${\lambda }_{2}^{*}$ is found especially for the two smaller venation networks from the Parkia and Schizolobium family. This result further emphasizes the intimate relation of dual communities and hierarchical organization in complex networks.

### Why do primal and dual communities emerge?

Understanding how the structure of optimal supply networks emerges is an important aspect of complex networks research^11,36–38^. In cases where a single source supplies the entire network, it is well established that fluctuations in the supply can cause a transition from a tree-like topology to a structure with loops^11,34,36^. We extend this result by studying how the increase in fluctuations influences the optimal network structure in supply networks with multiple strongly fluctuating sources and weakly fluctuating sinks. This design is highly relevant for many real-world applications, e.g., when considering a power grid that is based on decentralized renewable energy sources that fluctuate more than conventional carriers.

**Fig. 5:**
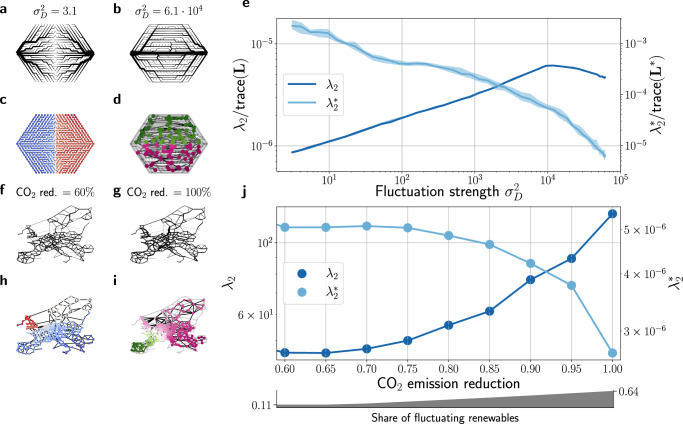
Primal and dual communities emerge naturally in optimal supply networks. **a**, **b** We consider a triangular lattice with two fluctuating sources located at the leftmost and the rightmost node and Gaussian sinks attached to all other nodes. The strength of the two sources fluctuates following a Dirichlet distribution with variance ${\sigma }_{D}^{2}$ (see “Methods”). For each value of ${\sigma }_{D}^{2}$, we find the network structure and the edge weights that minimize the total dissipated energy *D* assuming limited resources. **c**, **d** The optimal network structure shows a transition from primal to dual communities measured by the Fiedler vectors (color code) of the primal graph (**c**) or dual graph (**d**). **e** The scaling of the corresponding primal (*λ*_2_, dark blue) and dual (${\lambda }_{2}^{*}$, light blue) Fiedler value confirms the transition. The shaded regions indicate the 25–75% quantiles for different runs. **f**, **g** A transition from primal to dual community structure is also observed in optimization models of the European power grid when generation shifts to fluctuating renewables. The figure shows the optimal network structures and transmission line capacities in a cost optimal system for different carbon-dioxide (CO_2_) emission reduction levels. **h**, **i** Primal and dual communities are identified by the Fiedler vectors (color code) of (**h**) the primal or (**i**) dual graph. **j** The transition is confirmed by the scaling of primal and dual Fiedler values for increasing emission reduction which corresponds to an increasing share of fluctuating renewables (lower axis). Simulations were performed with the high-resolution European energy system model “PyPSA-EUR”^39^ that optimizes the investments and operations of generation and transmission infrastructures for minimum system costs.

To interpolate between strongly fluctuating sources and weakly fluctuating ones, we first use a similar model as in ref. 36. We consider a linear flow network consisting of a triangular lattice with *N* nodes of which *N*_*s*_ are sources and *N* − *N*_*s*_ are sinks whose outflows are fluctuating iid Gaussian random variables. Additionally, we add fluctuations only to the sources of the networks that can be tuned by the additional variance ${\sigma }_{D}^{2}$ (see “Methods”). We then compute the optimal structure and edge weights of the network that minimize the total dissipated energy $D={\sum }_{\ell }\langle {F}_{\ell }^{2}\rangle /{w}_{\ell }$ averaged over the fluctuating inflows and outflows. Resources for building the network are assumed to be limited, which translates into the constraint ${\sum }_{e}{w}_{e}^{\gamma }\le 1$. The cost parameter *γ* quantifies how expensive the increase of an edge weight is and was set to *γ* = 0.9 for the examples presented in this manuscript (see Supplementary Note 4 for more information). Results for *N*_*s*_ = 2 sources are shown in Fig. 5, and further results for *N*_*s*_ = 3 are provided in the Supplementary Information.

We find that the optimal network structure changes strongly as the fluctuations increase. For weak fluctuations, ${\sigma }_{D}^{2}\, \approx \,1$, each of the *N*_*s*_ sources supplies the surrounding area of the network. Only weak connections are established between the areas to cope with the small residual imbalances. Hence, the optimal networks show a pronounced primal community structure (see Fig. 5a).

For strong fluctuations, ${\sigma }_{D}^{2} \, \gg \, 1$, a local area supply is no longer possible and long-distance connectivity is required. Remarkably, this connectivity is established in one central vein that links the two fluctuating sources (see Fig. 5b). As a consequence, the optimal networks show a pronounced dual community structure similar to leaf venation networks. We can capture the transition from a primal to a dual community structure in terms of the primal and dual Fiedler values (Fig. 5e). Increasing ${\sigma }_{D}^{2}$, we observe a smooth crossover from primal communities with *λ*_2_ → 0 to dual communities with ${\lambda }_{2}^{*}\to 0$. We note that a similar picture is found if the Fiedler values *λ*_2_ and ${\lambda }_{2}^{*}$ are replaced by another measure such as the modularity (see Supplementary Fig. S1). We conclude that optimal supply networks typically have a community structure—whether it is primal or dual depends on the degree of fluctuations.

Strikingly, an analogous transition is observed for actual power transmission grids when optimizing the network structure for different levels of fluctuating renewable energy sources. We consider the European power transmission grid and optimize its network structure for different carbon dioxide (CO_2_) emission reduction targets compared to the year 1990 ranging from 60% to 100% reduction using the open energy system model “PyPSA-Eur”^39^ (see “Methods” for details). In Supplementary Figs. S3 and S4, we illustrate how the generation mix in the optimized power system changes for different emission scenarios from conventionally dominated grids to highly renewable grids.

We find that the decarbonization of power generation drives a transition from primal to dual communities in the grid. A reduction in generation-based CO_2_ emissions corresponds to an increased share of power being produced by fluctuating renewable energy sources. With increasing penetration of fluctuating renewables, we observe a decrease in the dual Fiedler value ${\lambda }_{2}^{*}$ and an increase in the primal Fiedler value *λ*_2_, which indicates a transition from primal to dual communities in the optimized networks (Fig. 5j). Hence, the primal-dual transition emerges both in fundamental models and in realistic high-resolutions simulations of spatial networks.

### How do primal and dual communities determine network robustness?

Primal and dual communities both impede the spreading of failures and thus improve the robustness of complex networks as shown in Fig. 1. We will now provide a more detailed and quantitative analysis of this connection for two important systems: flow networks and coupled oscillator networks.

We first consider linear flow networks using the theoretical framework introduced above. Robustness is quantified by a sensitivity factor, measuring the response of the network flows **F** to a perturbation. As a perturbation, we add an inflow Δ*P* at a node *v*_1_ and an outflow of the same amount at another node *v*_2_. Here, we focus on the case where *v*_1_ and *v*_2_ are the two end nodes of an edge *e* = (*v*_1_, *v*_2_) and treat the general case in the Supplementary Information. The source vector in the Poisson Eq. (2) then changes as

13 $${{{{{{{\bf{P}}}}}}}}\to {{{{{{{\bf{P}}}}}}}}^{\prime}={{{{{{{\bf{P}}}}}}}}+{{\Delta }}P\,{{{{{{{{\bf{I}}}}}}}}}^{\top }{{{{{{{{\bf{l}}}}}}}}}_{e}$$

and ${{{{{{{{\bf{l}}}}}}}}}_{e}\in {{\mathbb{Z}}}^{M}$ is the indicator function for edge *e*, which is equal to one at the positions indicated by the subscript and zero otherwise. Inverting the discrete Poisson Eq. (2), we then find that the network flows change by the amount

14 $${{\Delta }}{{{{{{{\bf{F}}}}}}}}={{\Delta }}P\,{{{{{{{\bf{W}}}}}}}}{{{{{{{\bf{I}}}}}}}}{{{{{{{{\bf{L}}}}}}}}}^{{{{\dagger}}} }{{{{{{{{\bf{I}}}}}}}}}^{\top }{{{{{{{{\bf{l}}}}}}}}}_{e},$$

where **L**^†^ is the Moore–Penrose pseudoinverse of the primal graph Laplacian. We then define a sensitivity factor as the ratio of the flow change at edge *ℓ* and the perturbation strength Δ*P* as^40,41^

15 $${\eta }_{{v}_{1},{v}_{2},\ell }=\frac{{{\Delta }}{F}_{\ell }}{{{\Delta }}P}={w}_{\ell }{{{{{{{{\bf{l}}}}}}}}}_{\ell }^{\top }{{{{{{{\bf{I}}}}}}}}{{{{{{{{\bf{L}}}}}}}}}^{{{{\dagger}}} }{{{{{{{{\bf{I}}}}}}}}}^{\top }{{{{{{{{\bf{l}}}}}}}}}_{e}.$$

We note that the sensitivity factor is widely used in the context of power system security analysis, where it is referred to as a power transfer distribution factor^40,41^. Importantly, the sensitivity factor may also be used to simulate the failure of an edge *e* = (*v*_1_, *v*_2_) by choosing the inflow Δ*P* accordingly (see Supplementary Information).

The sensitivity factor ${\eta }_{{v}_{1},{v}_{2},\ell }$ elucidates the relation between primal communities and network robustness^8^. In the Supplementary Information, we treat the limiting case of vanishing connectivity between the communities and show the following: If the edges *e* and *ℓ* are in different communities, *η* vanishes in the same way as the Fiedler value *λ*_2_. If the edges *e* and *ℓ* are in the same community, *η* remains finite as *λ*_2_ → 0.

Remarkably, we can find an analogous description in the dual graph^19,20^. In Eq. (4), we choose the particular solution as Δ*F*_part_ = Δ*P* **l**_*e*_. We can then compute the cycle flows **f** from Eq. (8) and substitute the result into Eq. (6) to obtain the change of network flows^19,20^

16 $${{\Delta }}{{{{{{{\bf{F}}}}}}}}=-\!\!{{\Delta }}P\,{{{{{{{\bf{C}}}}}}}}{({{{{{{{{\bf{L}}}}}}}}}^{*})}^{{{{\dagger}}} }{{{{{{{{\bf{C}}}}}}}}}^{\top }{{{{{{{{\bf{W}}}}}}}}}^{-1}{{{{{{{{\bf{l}}}}}}}}}_{e}+{{\Delta }}P\,{{{{{{{{\bf{l}}}}}}}}}_{e}.$$

The sensitivity factor for all edges *ℓ* ≠ *e* thus reads

17 $${\eta }_{{v}_{1},{v}_{2},\ell }=-\frac{1}{{w}_{e}}{{{{{{{{\bf{l}}}}}}}}}_{\ell }^{\top }{{{{{{{\bf{C}}}}}}}}{({{{{{{{{\bf{L}}}}}}}}}^{*})}^{{{{\dagger}}} }{{{{{{{{\bf{C}}}}}}}}}^{\top }{{{{{{{{\bf{l}}}}}}}}}_{e}.$$

We see that the dual Laplacian L^*^ contributes to the sensitivity factor ${\eta }_{{v}_{1},{v}_{2},\ell }$ in exactly the same way as the primal Laplacian **L** in Eq. (15). Hence, we conclude that primal and dual community structures determine network flows in an equivalent manner. If the edges *e* and *ℓ* are in different dual communities, *η* will vanishes proportional to the dual Fiedler value ${\lambda }_{2}^{*}$. If the edges *e* and *ℓ* are in the same community, *η* remains finite in the limit ${\lambda }_{2}^{*}\to 0$.

**Fig. 6:**
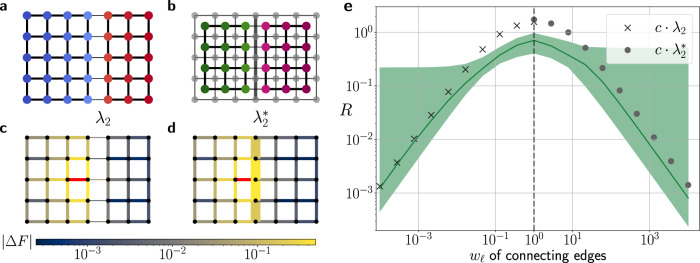
Primal and dual communities inhibit failure spreading. **a**, **b** A square grid is divided into **a** two primal communities by weakening the central horizontal edges or **b** into two dual communities by strengthening the central vertical edges. The Fiedler vector (color code) reveals the community structure. **c**, **d** Both primal and dual communities inhibit flow changes ∣Δ*F*∣ (color coded) in the other community after the failure of a single edge (red) with unit flow in a given community. **e** We interpolate between primal and dual communities in a square grid of size 21 × 10 by tuning the weight *w*_*e*_ of the horizontal edges or vertical edges (see **a**, **b**). The flow ratio *R* reveals that failure spreading to the other community is largest for *w*_*ℓ*_ = 1. It decays for either type of community as measured by primal and dual Fiedler values *λ*_2_ (crosses) and ${\lambda }_{2}^{*}$ (circles), respectively. The green line represents the median value and the shaded regions indicate the 25% and 75% quantiles. The symbol *c* denotes a normalization constant used to improve comparison with *R*.

We now quantify this effect. To analyze the impact of a community structure, we consider a square lattice with tunable edge weights. We either reduce the edge weights *w*_*ℓ*_ across the boundary, i.e., in the cut-set, to induce a primal community structure, or we increase the edge weights *w*_*ℓ*_ along the boundary, i.e., in the cut-path, to induce a dual community structure (Fig. 6a, b). We then consider an inflow and simultaneous outflow Δ*P* at two nodes *v*_1_ and *v*_2_, respectively, that are connected via an edge *e* = (*v*_1_, *v*_2_). We then compare the resulting flow changes in the same (S) and the other (O) community as the given edge *e*. To this end, we evaluate the ratio of flow changes *R*(*e*, *d*) in the two communities at a given distance *d* to the trigger edge *e*^33^

18 $$R(e,d)=\frac{{\langle|{{\Delta }}{F}_{k}|\rangle }_{d}^{k\in {{{{{{{\rm{O}}}}}}}}}}{{\langle|{{\Delta }}{F}_{r}|\rangle }_{d}^{r\in {{{{{{{\rm{S}}}}}}}}}}=\frac{{\langle|{\eta }_{{v}_{1},{v}_{2},k}|\rangle }_{d}^{k\in {{{{{{{\rm{O}}}}}}}}}}{{\langle|{\eta }_{{v}_{1},{v}_{2},r}|\rangle }_{d}^{r\in {{{{{{{\rm{S}}}}}}}}}}.$$

Here, ${\langle \cdot \rangle }_{d}^{\ell \in C}$ denotes the average over all edges *ℓ* in a community *C* at a given distance *d* to the trigger edge *e*. To be able to neglect the effect of a specific edge and the distance, we average over all possible trigger edges *e* and distances *d* to arrive at the mean flow ratio

19 $$R={\langle R(e,\, d)\rangle }_{e,d}.$$

The mean flow ratio ranges from *R* ≈ 0 if the other module is weakly affected, i.e., there is a strong community effect, to *R* ≈ 1 if there is no noticeable effect. We note that *R* describes flow changes after perturbations in the inflows and outflows as well as flow changes as a result of the complete failure of edges (see Supplementary Information).

Figure 6 illustrates that both primal and dual communities suppress flow changes in the other community. The mean flow ratio *R* decays for either community structure. In particular, this decay is well-captured by the Fiedler value of the primal (*λ*_2_) and the dual (${\lambda }_{2}^{*}$) graph.

These findings are not restricted to linear flow networks, but hold for all diffusively coupled networked systems. We illustrate this effect for a network of second-order phase oscillators that arises in the analysis of electric power grids^18,42^ or mechanically coupled systems^43^ and as a generalization of the celebrated Kuramoto model^44^. The phase *ϑ*_*i*_(*t*) of each oscillator *i* = 1, …, *N* evolves according to

20 $${M}_{i}{\ddot{\vartheta }}_{i}+{D}_{i}{\dot{\vartheta }}_{i}={\omega }_{i}+\mathop{\sum }_{j=1}^{N}{w}_{ij}\sin ({\vartheta }_{j}-{\vartheta }_{i}),$$

where *M*_*i*_ is the inertia and *D*_*i*_ the damping of the *i*th oscillator. To analyze the impact of community structures, we consider a honeycomb lattice with tunable edge weights, with either low weights *w*_*ij*_ ≤ 1 across the boundary or high weights *w*_*ij*_ ≥ 1 along the boundary. The weights of all remaining edges are set to *w*_*ij*_ = 1 and *w*_*ij*_ = 0 if no edge exists between nodes *i* and *j*.

**Fig. 7:**
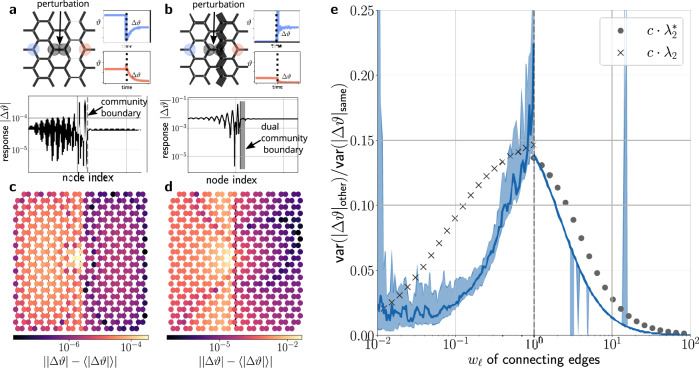
Suppression of failure spreading in oscillator networks. **a**, **b** We analyze the response of a network of phase oscillators (Eq. (20)) to a localized perturbation. We consider a honeycomb lattice with **a** a primal community structure induced by weak connectivity across the community boundary and **b** a dual community structure induced by strong connectivity along the boundary. After the perturbation the oscillators relax back to a phase-locked state with phases shifted by Δ*ϑ*_*i*_. **c**, **d** We find that the response ∣Δ*ϑ*_*i*_∣ is strongly suppressed in the non-perturbed community. **e** The overall network response is quantified by the variance of the response within a network community (Eq. (21)). The response in the non-perturbed community is strongly suppressed—the more pronounced the community structure, the stronger the attenuation. The existence of a primal or dual community structure is indicated by primal and dual Fiedler values *λ*_2_ (crosses) and ${\lambda }_{2}^{*}$ (circles), respectively. The blue line represents the median value and the shaded regions indicate the 25 and 75% quantiles for different random realizations of the natural frequencies *ω*_*i*_. The symbol *c* denotes a normalization constant used to improve comparison with the response.

We now investigate how the steady states of such a network react to a localized perturbation near the community boundary (Fig. 7a, b). The oscillators relax to a phase-locked state after a short transient period, but the steady-state phases are shifted by an amount Δ*ϑ*_*i*_. We recall that a global phase shift is physically irrelevant and is henceforth discarded. The response ∣Δ*ϑ*_*i*_∣ crucially depends on the location of the oscillator—being strongly suppressed across the community boundary (Fig. 7a–d). To evaluate the impact of the network structure, we quantify the overall network response by the variance of the phases within a community *C*,

21 $${{{{{{{\rm{var}}}}}}}}\left(|{{\Delta }}{\vartheta }_{i}{|}_{C}\right)=\mathop{\sum }_{i\in C}|{{\Delta }}{\vartheta }_{i}{|}^{2}-{\left(\mathop{\sum }_{i\in C}|{{\Delta }}{\vartheta }_{i}|\right)}^{2}.$$

This overall response is generally suppressed in the non-perturbed community, for primal as well as for dual communities. The more pronounced the community structure, the stronger the suppression of the response (Fig. 7e). We note that for the current example some differences exist between primal and dual communities. In particular, statistic fluctuations are larger in the case of primal communities.

We conclude that the impact of community boundaries, both primal and dual, extends to all diffusively coupled networked systems. Our finding can be further substantiated by a linear response analysis^8^, which highlights the structural similarity to linear flow networks. Furthermore, we note that related phenomena were observed for models of information diffusion in networks of different modularity^25^. This finding is closely related, as the diffusion model includes an averaging over all adjacent nodes in the network.

## Discussion

We have introduced a way to define and identify dual communities in planar graphs. We demonstrated that both primal and dual community structures emerge as different phases of optimized networks – whether the one or the other is realized in a given optimal network depends on the degree of fluctuations. In addition to that, both types of communities have the ability to suppress failure spreading. They are thus optimized to limit the effect of edge failures or other perturbations.

An important difference between primal and dual communities is the fact that the former are based on a weak connectivity, while dual communities require a strong connectivity. This has significant consequences for supply networks such as power grids. Several approaches have been discussed to limit the connectivity of power grids to prevent the spreading of cascading failures. This includes concepts of microgrids^45^ as well as intentional islanding^46^ or tree-partitioning^47,48^. However, future power grids will require more, not less connectivity to transmit renewable energy over large distances ^49,50^. Dual communities might resolve this conundrum, as they prevent failure spreading from one community to the other one, without limiting the network’s ability to transmit energy. This is in stark contrast to primal communities that limit failure spreading from one community to the other one, but also supply. Thus, the construction of dual communities may also serve as a strategy against failure spreading, in line with other ideas brought forward recently^33^.

Dual communities may be detected using the same techniques as for primal communities once the dual graph is constructed. We here focus on classical spectral methods based on the graph Laplacian, as this matrix naturally arises in the study of graph duality and linear flow networks. By now, numerous algorithms for community detection have been developed that outperform spectral methods depending on the respective application^5,51,52^. All these algorithms can be readily applied to the dual graph. A short analysis for a selected example is provided in the Supplementary Information. One challenge remains for the generalization of this approach. For planar graphs, the dual is constructed by a straightforward geometric procedure. For non-planar graphs, a geometric analysis is much more involved^53^. A dual can be constructed algebraically by choosing a basis of the cycle space. However, there is no distinguished basis such that the algebraic dual is not unique. The detailed analysis of community boundaries, in particular the inequality (Eq. (12)), may provide an alternative route to generalize the definition of network communities. For instance, one may choose a decomposition to minimize the dual topological connectivity ${\mu }_{2}^{*}$.

Finally, we note that other approaches have been put forward to generalize the definition of network communities beyond the paradigm of strong mutual connectivity. For instance, communities can be defined in terms of the similarity of the connectivity of nodes (see, e.g., refs. 54, 55) or from spreading processes^56^. The graph dual approach presented here emphasizes the role of the community boundaries, both in the definition of the community structure and in its impact on spreading processes and network robustness. Furthermore, graph duality provides a rigorous algebraic justification for our generalization of community structures.

## Methods

### Global cascade model

In Fig. 1, we show results from a classic model of global cascades. The state of each node *i* = 1, …, *N* in time step *t* is denoted as *s*_*i*_(*t*) ∈ {0, 1}, encoding healthy/operational and infected/faulty, respectively. A node becomes infected/faulty in time step *t* + 1 if the weighted average of the neighboring nodes exceeds a threshold *ϕ*_*i*_:

22 $${s}_{i}(t+1)=\left\{\begin{array}{lll}1&{{{{{{{\rm{if}}}}}}}}&\frac{{\sum }_{k}{w}_{ik}{s}_{k}}{{\sum }_{k}{w}_{ik}} \, > \, {\phi }_{i}\\ 1&{{{{{{{\rm{if}}}}}}}}&{s}_{i}(t)=1\\ 0&&{{{{{{{\rm{else}}}}}}}}.\end{array}\right.$$

This model is iterated until no further changes of the node states occur.

We simulate this model on a square lattice with inhomogeneous edge weights. A fraction *p*_*e*_ = 0.8 of edges connecting the center nodes of the lattice with its nearest neighbors is selected at random. The weight of these edges is set to *w*_*ij*_ = *w*_*ℓ*_, where *w*_*ℓ*_ is a tunable parameter, while all other edges have weight *w*_*ij*_ = 1. At time *t* = 0, we choose a fraction *ρ*_0_ = 0.05 of all nodes in the left part and set them to state 1, while all other nodes are in state 0. For each value of the parameter *w*_*ℓ*_, we repeat the simulation for 1000 random initial conditions and record the fraction of nodes in state 1, denoted as *ρ*_*∞*_.

### Creation of dual graphs: planar networks

In this manuscript, we mostly restrict our analysis to planar, connected graphs. A graph *G* = (*V*, *E*) with vertex set *V* and edge set *E* is called planar if it may be drawn in the plane without two edges crossing^57^. For a plane graph *G*, it is straightforward to establish a duality to another graph, referred to as the plane dual or simply dual graph and denoted as G^*^. The dual graph is constructed using the cycles of graph *G* where a cycle is defined to be a path that starts and ends in the same vertex consisting of otherwise distinct vertices. For a graph with *M* edges and *N* nodes, these cycles form the graph’s cycle space of dimension N^*^ = *M* − *N* + 1. A particular basis of this space is given by the faces of the plane embedding, such that the dual graph G^*^ = (V^*^, E^*^) has a vertex corresponding to each face. Two dual vertices ${v}_{1}^{*}$ and ${v}_{2}^{*}$ are connected by a dual edge ${e}^{*}=({v}_{1}^{*},\, {v}_{2}^{*})\in {E}^{*}({G}^{*})$ if the two corresponding cycles share an edge. For a weighted graph, the edge weight of the dual edge is chosen to be the inverse of the corresponding edge shared by the two cycles. Furthermore, we adopt the following convention; if two cycles share *k* edges *e*_1_, . . , *e*_*k*_ with weights *w*_1_, . . . , *w*_*k*_, we lump them together into a single dual edge e^*^ with edge weight ${w}^{*}=\mathop{\sum }\nolimits_{i=1}^{k}{w}_{i}^{-1}$ thus avoiding multi-edges in the dual graph and refer to this model as the reduced dual graph. Note that the definition of the edge-cycle incidence matrix **C** needs to be adjusted for the reduced dual graph.

### Creation of dual graphs: non-planar networks

For non-planar networks, the basis of the cycle space may no longer be uniquely determined based on the graph’s embedding. Different basis choices result in different dual graphs. When calculating the dual graph of the non-planar European topology shown in Fig. 5f–g, we used the graphs’ minimum cycle basis to create the dual graph.

### Hierarchical decomposition of dual graph

We assign *m* hierarchy levels based on repeated spectral bisection of the dual graph using the following procedure:

1. Assign dual communities to the graph by making use of the Fiedler vector ${{{{{{{{\bf{v}}}}}}}}}_{2}^{*}$ of the dual graph G^*^
2. Identify the edges that lie on the boundary between the two communities by checking for edges in the primal shared by faces corresponding to dual nodes of both communities
3. Remove the boundary edges from the graph thus creating two primal subgraphs *G*_1_ and *G*_2_
4. Repeat the process *m* times

### Building supply networks with fluctuating sources

Our framework extends the fluctuating sink model proposed by Corson^36^ where a single, fluctuating source supplies the remaining network. To this end, we consider a linear flow network with sources and sinks attached to the nodes and model the sinks as Gaussian random variables $P\in {{{{{{{\mathcal{N}}}}}}}}(\mu,\, \sigma )$. In contrast to previous work, we consider multiple sources, *N*_*s*_ in number, whose statistics can be derived from the statistics of the sinks due to the fact that the in- and outflows at the nodes need to sum to zero (see Supplementary Information). We then add additional fluctuations to the sources that are built using Dirichlet random variables *X*_*i*_ ~ Dir(*α*). The fluctuations are constructed such that they only influence the statistics of the sources and their variance is tuned by a single parameter *α*. To be able to tune the influence of this additive noise variable, we introduce a scale parameter $K\in {\mathbb{R}}$. The inflow at a source at a given point in time is then given by (see Supplementary Information)

23 $${P}_{{s}_{i}}=-\frac{1}{{N}_{s}}\mathop{\sum }_{i={N}_{s} \!+1}^{N}{P}_{i}+K\left(\frac{1}{{N}_{s}}-{X}_{i}\right),$$

where *P*_*i*_ are the outflows at the sinks. Here, we arranged the node order such that the sources have indices 1, …, *N*_*s*_ and sinks are numbered as *N*_*s*_ + 1, …, *N*. To produce Fig. 5, we considered a network with *N*_*s*_ = 2 and fix the parameters of the Gaussian distribution as *μ* = −1, *σ* = 0.1. The scale parameter is set to *K* = 500 and the parameter *α* controlling the statistics of the Dirichlet distribution is varied in the interval *α* ∈ [10^−2^, 10^4^], thus changing the variance of the Dirichlet variables ${\sigma }_{D}^{2}={K}^{2}\frac{({N}_{s}-1)}{{N}_{s}^{2}({N}_{s}\alpha+1)}$ (see Supplementary Information).

### Analysis of power grid datasets

The networks shown in Fig. 5f, g were determined using the open energy system model ’PyPSA-Eur’ cost-optimizing the generation infrastructure and operation as well as the transmission grid for different levels of carbon-dioxide emission reductions with respect to the emission levels in 1990. For each target carbon-dioxide emission reduction level, the network is optimized for an entire year with the weather conditions of 2013 and 3-hourly resolution (see ref. 39 for further details on the optimization model). To analyze the network topology, we set the weight *w*_*ℓ*_ of a line *ℓ* to the maximal apparent power that can flow through it. Note that this is different from weighting the line by its line susceptance and allows us to also incorporate high-voltage DC lines. To determine the level of fluctuating renewables shown in Fig. 5f, g, we calculate the share of the total annual generation in the entire system that is produced by fluctuating renewables. To this end, we assume that the following technologies are fluctuating renewable energy sources: offshore wind AC, offshore wind DC, onshore wind, run-of-the-river hydroelectricity (ror) and solar. In Supplementary Figs. S3 and S4 we show as an example the generation for two months and carbon emission reduction levels over time and on the network level.

## Supplementary information

Supplementary Information

## Data Availability

The topology of the Central European power grid have been extracted from the open European energy system model PyPSA-Eur^39^, which is fully available online^58^. Leaf data was provided by the authors of ref. 59 and is available from the respective authors upon request. The leaf venation networks are based on microscopic recordings. Edge conductivities *w*_*i**j*_ are assumed to scale with the radius *r*_*i**j*_ of the corresponding vein (*i*, *j*) as ${w}_{ij}\propto {r}_{ij}^{4}$ according to the Hagen-Poisseuille law (see ref. 60 for a detailed discussion). We used the radius in pixels at a resolution of 6400 dpi. The data generated in this study (effective topology of power grid networks and selected leaf venation networks) as well as essential computer code for data processing have been deposited in a Zenodo repository^61^.

## Code availability

Computer code is available on github^62^ with the specific version used in this publication being archived at Zenodo^61^.
