# Supplementary material for: Dual communities in spatial networks
Source: Nat Commun. 2022 Dec 3;13:7479. doi: 10.1038/s41467-022-34939-6 (PMC9719545; doi:10.1038/s41467-022-34939-6)
Supplement: Supplementary file 1 — Supplementary Information [file 41467_2022_34939_MOESM1_ESM.pdf]

Supplementary Information for
Dual communities in spatial networks

Franz Kaiser,[1,2] Philipp C. Böttcher,[1] Henrik Ronellenfitsch,[3,4] Vito Latora,[5,6,7] and Dirk Witthaut[1,2]

[1]*Forschungszentrum Jülich, Institute for Energy and Climate Research (IEK-STE), 52428 Jülich, Germany*
[2]*Institute for Theoretical Physics, University of Cologne, Köln, 50937, Germany*
[3]*Physics Department, Williams College, 33 Lab Campus Drive, Williamstown, MA 01267, U.S.A.*
[4]*Department of Mathematics, Massachusetts Institute of Technology, Cambridge, MA 02139, U.S.A.*
[5]*School of Mathematical Sciences, Queen Mary University of London, London E1 4NS, UK*
[6]*Dipartimento di Fisica ed Astronomia, Università di Catania and INFN, 95123 Catania, Italy*
[7]*Complexity Science Hub Vienna, 1080 Vienna, Austria*

(Dated: October 26, 2022)

This Supplementary Material contains five Supplementary Notes and six Supplementary Figures.

Supplementary Figures 3

Supplementary Notes 8
Supplementary Note 1: Graph duality 8
Supplementary Note 2: Algebraic and topological connectivity 12
Supplementary Note 3: Linear flow networks 16
Supplementary Note 4: Fluctuating sink model with additive Dirichlet noise 22
Supplementary Note 5: Clustering in primal and dual graphs 24

Supplementary References 25

SUPPLEMENTARY FIGURES

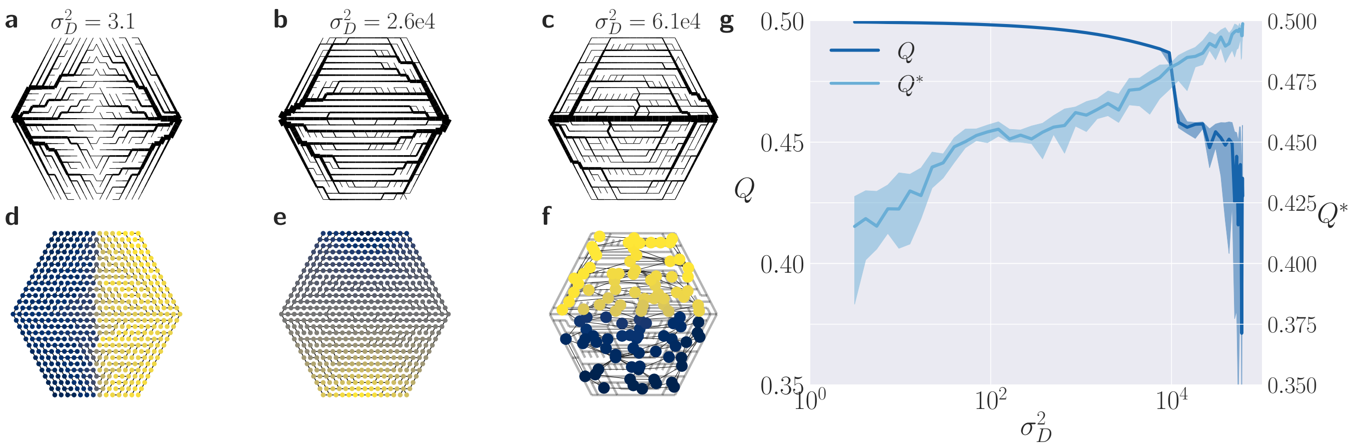

Supplementary Figure 1. **Primal and dual communities emerge naturally in optimal supply networks. a-c,** We consider a triangular lattice with two fluctuating sources located at the leftmost and the rightmost corner. The remaining nodes correspond to sinks, whose demand fluctuates following an iid normal distribution. The sources balance the demand in total, but the spatial distribution varies according to a Dirichlet distribution introducing additional fluctuations with variance σ_D^2. We then determine the optimal network structure and edge weights to minimise the average dissipation [1]. We observe a transition from (**a**) a primal community structure at low fluctuations to (**c**) a dual community structure at high fluctuations. **d-f,** Communities can be identified according to the Fiedler vector. The figure shows (**d,e**) the primal Fiedler vector and (**f**) the dual Fiedler vector in a colour map for the networks depicted in panels (**a-c**), respectively. **g,** The transition from a primal to a dual community structure can be quantified either by the algebraic connectivity (cf. main text) or by the modularity [2]. The figure shows the modularity Q of the primal community decomposition and the modularity Q^* of the dual community decomposition as a function of the variance σ_D^2 of the source strength.

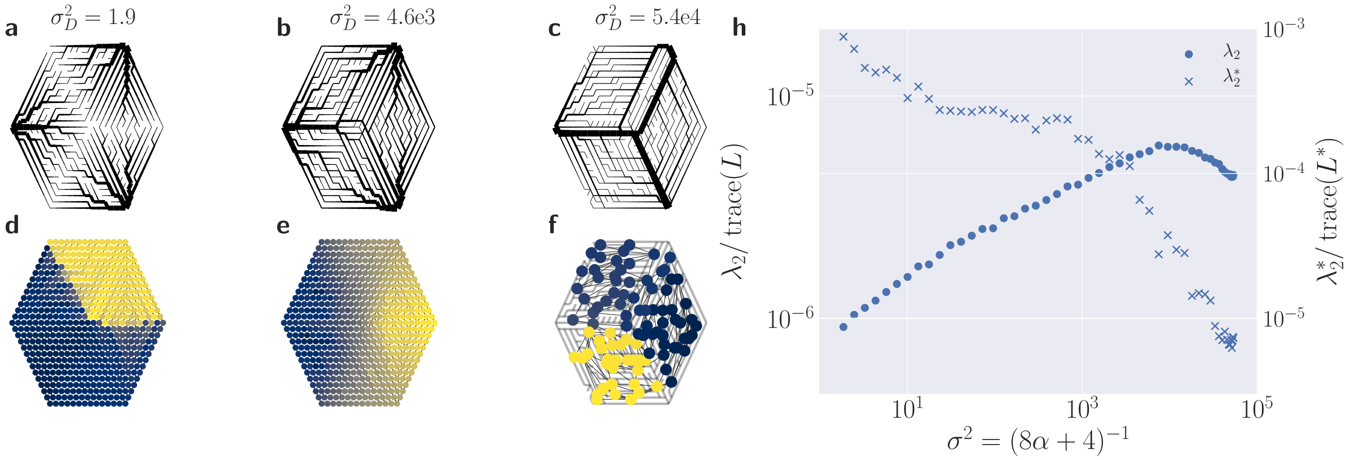

Supplementary Figure 2. **Primal and dual communities emerge naturally in optimal supply networks with three sources. a-c,** We consider a triangular lattice with three fluctuating sources located at the leftmost, the upper right and the lower right corner. The remaining nodes correspond to sinks, whose demand fluctuates following an iid normal distribution. The sources balance the demand in total, but the spatial distribution varies according to a Dirichlet distribution introducing additional fluctuations with variance σ_D^2. We then determine the optimal network structure and edge weights to minimise the average dissipation [1]. We observe a transition from (**a**) a primal community structure at low fluctuations to (**c**) a dual community structure at high fluctuations. **d-f,** Communities can be identified according to the Fiedler vector. The figure shows (**d,e**) the primal Fiedler vector and (**f**) the dual Fiedler vector in a colour map for the networks depicted in panels (**a-c**), respectively. **h,** The scaling of the corresponding primal (λ_2, circles) and dual (λ_2^*, crosses) Fiedler value confirms the transition.

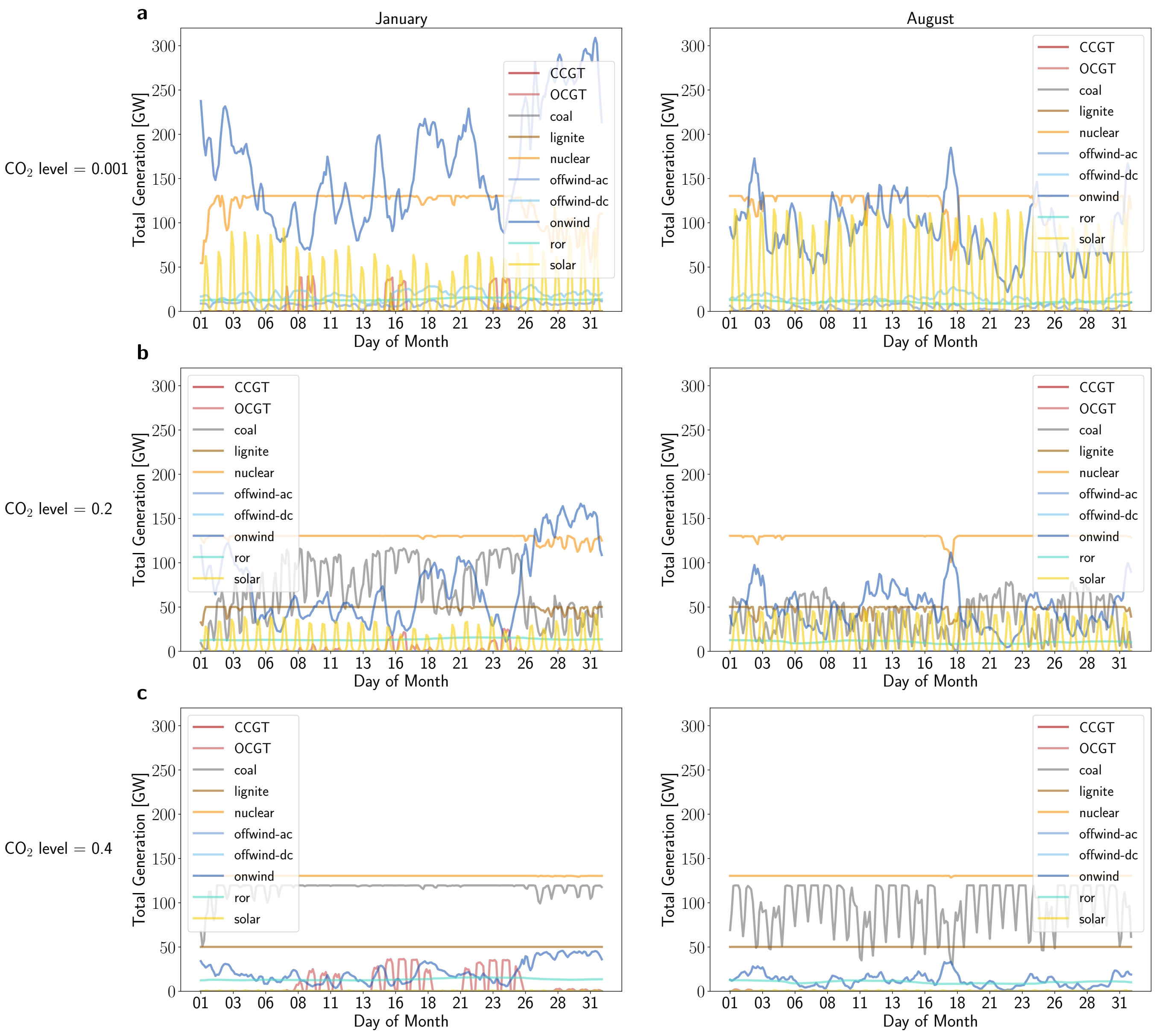

Supplementary Figure 3. **Optimized electric power systems simulated with the open energy system model 'PyPSA-Eur'.** The model optimizes both the investments and the operation of the generation and transmission infrastructures to minimize the total system costs for a given CO_2 cap. The figure shows the total power generation by generator type for two exemplary months in winter (January, left) and summer (August, right) with a temporal resolution of 2 hours. Results are shown for three different values of the CO_2 level measured relative to the reference year 1990: (**a**) 0.001, (**b**) 0.2, and (**c**) 0.4. With decreasing levels of CO_2, generation shifts more and more towards fluctuating renewable power sources.

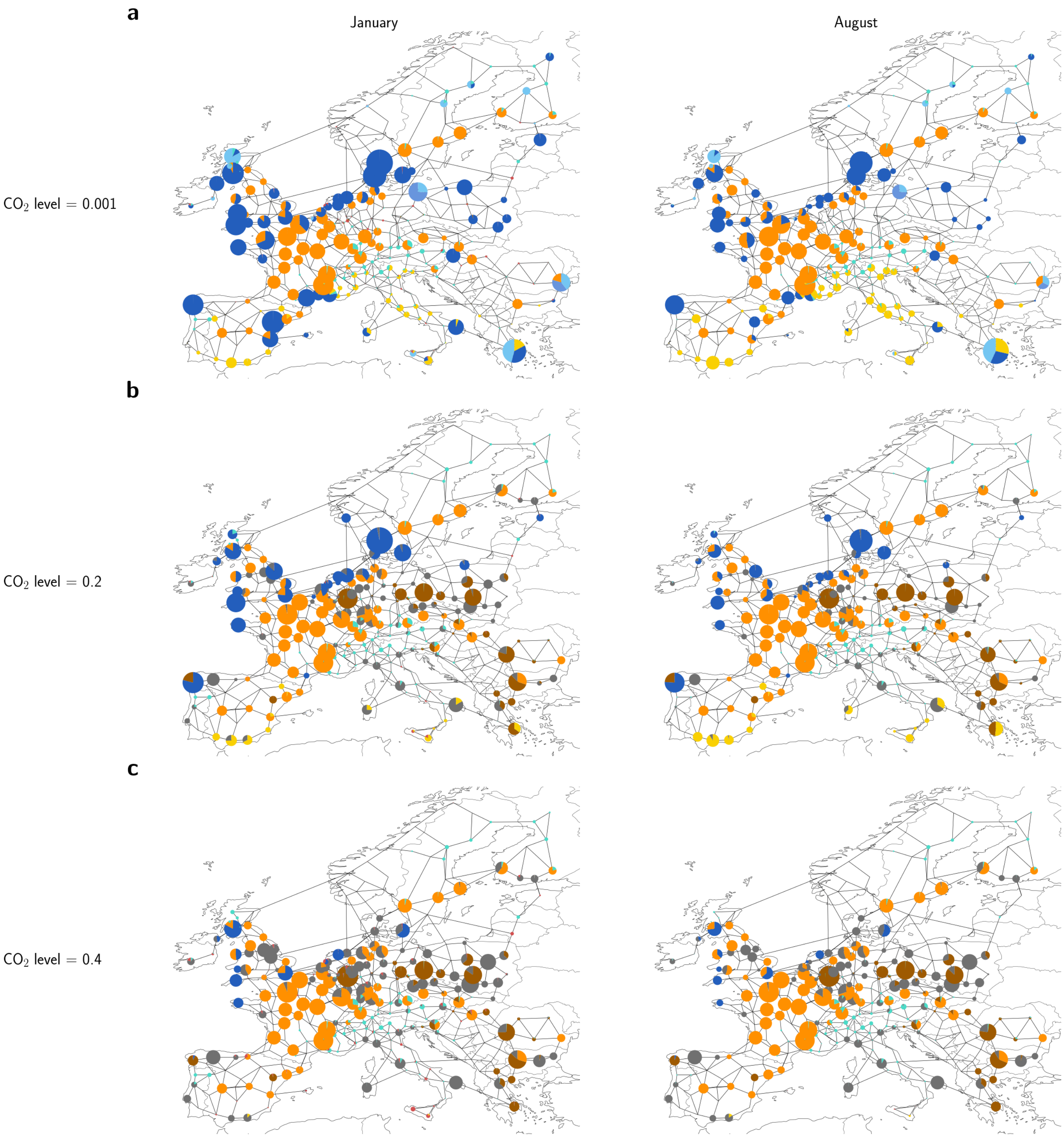

Supplementary Figure 4. **Electric power systems and grids during the energy transition.** The figure shows the change in power generation and transmission infrastructures as the CO_2 level is reduced. Power system layout and operation are optimized using the open energy system model 'PyPSA-Eur'. The figure shows the total generation per month and grid node for two exemplary months in winter (January, left) and summer (August, right) as well as the layout of the power transmission grid. Generation is shown in a pie chart, where the size is proportional to the total generation and the colour code indicates the generation type, cf. Supplementary Figure 3. Results are shown for three different values of the CO_2 level measured relative to the reference year 1990: (**a**) 0.001, (**b**) 0.2, and (**c**) 0.4. With decreasing levels of CO_2, generation shifts more and more towards fluctuating renewable power sources with pronounced regional differences: Wind power in predominantly generated along the coast, especially around the North Sea. Solar power is generated predominantly in Southern Europe.

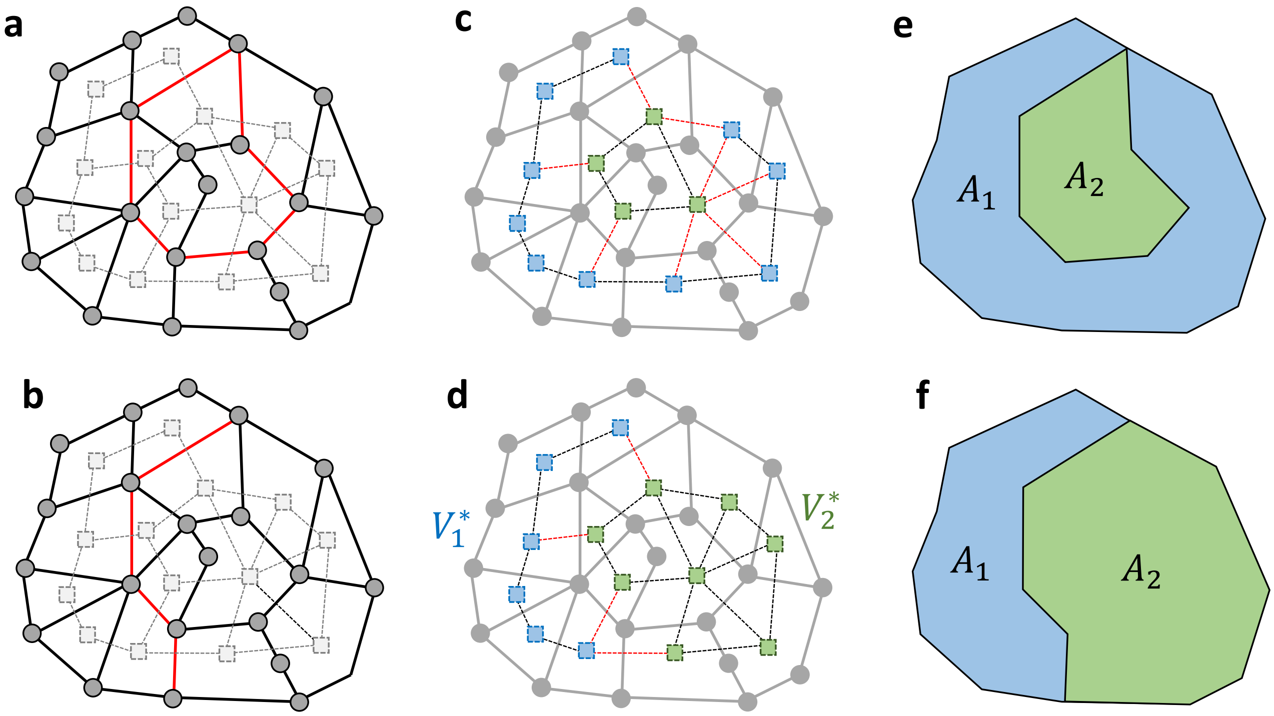

Supplementary Figure 5. **Definition of a cut-path. a,b,** A graph (solid lines) and its weak dual (dashed lines). The edge set of a cut-path S is indicated in red colour. **c,d,** The cut-path induces a cut of the dual graph into two connected vertex sets V_1^* (blue) and V_2^* (green). The set S^* is a minimal cut set in the dual graph. **e,f,** The faces corresponding to the vertex sets V_1^* and V_2^* form two connected polygons A_1 and A_2 in the plane.

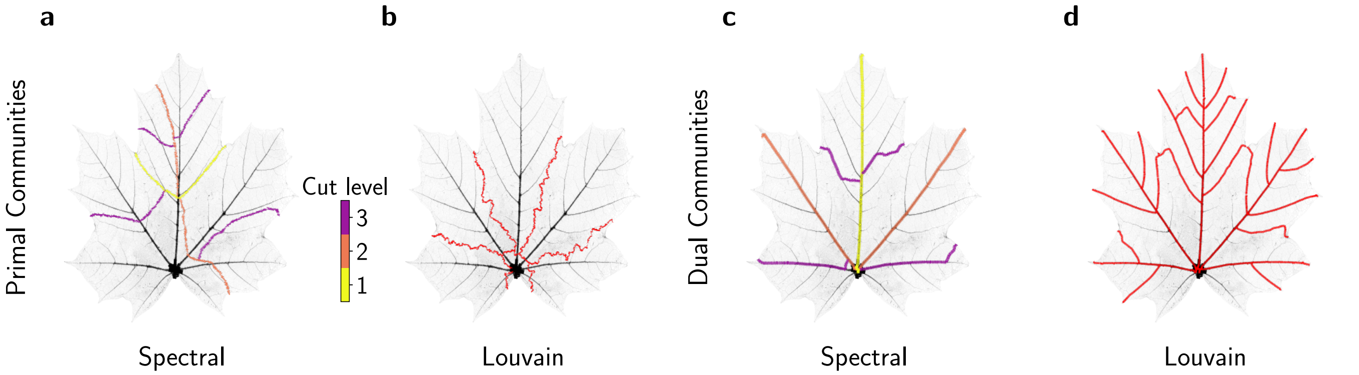

Supplementary Figure 6. **Different clustering methods in primal and dual graphs** Distinct primal and dual communities can be found using both spectral clustering and the *Louvain Detection Algorithm*. The original (i.e. primal) leaf venation network of *Acer platanoides* is shown in black in each panel. The resulting hierarchical cuts for three cut levels employing spectral clustering using the primal graph Laplacian and the dual graph Laplacian can be seen in panel **a** and **c**. The community structure revealed by the *Louvain Detection Algorithm*, choosing a resolution parameter $\xi = 0.01$, using the primal graph and dual graph are shown in panel **b** and **d**, respectively.

SUPPLEMENTARY NOTES

Supplementary Note 1: Graph duality

In this note, we review the fundamental definitions and properties of dual graphs extending the main text.

Basic definitions

Let $G = (V, E)$ be a plane, undirected graph with $N = |V|$ vertices and $L = |E|$ edges. We label all vertices consecutively as $(1, 2, 3, \dots, N)$. Edges are either referred to in terms their respective endpoints or by consecutive labels as $(1, 2, 3, \dots, L)$. We begin with unweighted graphs and include edge weights later. For each edge, we fix an orientation – for instance to keep track about the direction of flows as discussed below. The topology of the graph and the orientations are encoded in the node-edge incidence matrix $\mathbf{I} \in \mathbb{R}^{L\times N}$ with entries [3]

$$I_{\ell,n} = \begin{cases} 1 & \text{if edge } \ell \text{ starts at node } n, \\ -1 & \text{if edge } \ell \text{ ends at node } n, \\ 0 & \text{otherwise.} \end{cases}$$

The Graph Laplacian is defined as

$$\mathbf{L} = \mathbf{I}^\top \mathbf{I}. \tag{1}$$

This definition coincides with the definition

$$\mathbf{L} = \mathbf{D} - \mathbf{A},$$

in terms of the adjacency matrix $\mathbf{A} \in \mathbb{R}^{N\times N}$ with entries

$$A_{m,n} = \begin{cases} 1 & \text{if nodes } m \text{ and } n \text{ are connected,} \\ 0 & \text{otherwise.} \end{cases}$$

and the diagonal degree matrix $\mathbf{D} \in \mathbb{R}^{N\times N}$ with entries

$$D_{n,n} = \sum_m A_{n,m}.$$

A cycle in the graph can be described by a vector $\vec{v} \in \mathbb{R}^L$ with entries

$$v_\ell = \begin{cases} +1 & \text{if edge } \ell \text{ is part of the cycle,} \\ -1 & \text{if the reversed edge } \ell \text{ is part of the cycle,} \\ 0 & \text{otherwise.} \end{cases}$$

that satisfies

$$\mathbf{I}\vec{v} = \vec{0}. \tag{2}$$

This property is easy to understand for flow networks: Here, the vector $\vec{v}$ describes a cycle flow which has neither sink or source such that $\mathbf{I}\vec{v} = \vec{0}$. The cycle flows constitute a vector space that coincides with the kernel of the edge incidence matrix $\mathbf{I}$. In plane graphs, the faces provide a distinguished basis of the cycle space. This basis is encoded in the cycle incidence matrix $\mathbf{C}$ with entries

$$C_{\ell,f} = \begin{cases} 1 & \text{if the edge } \ell \text{ is part of face } f, \\ -1 & \text{if the reversed edge } \ell \text{ is part of face } f, \\ 0 & \text{otherwise.} \end{cases}$$

For convenience, we fix the orientation of each face in the counter-clockwise direction.

We now proceed to the definition of the dual graph $G^* = (V^*, E^*)$. Two common definitions exist, differing in the treatment of the exterior of the graph.

1. The vertex set V^* consists of all faces of the primal graph, including the outer face. Then, the dual graph has $|V^*| = L - N + 2$ vertices.

2. The vertex set V^* consists of all faces of the primal graph, excluding the outer face. Then, the dual graph has $|V^*| = L - N + 1$ vertices. This graph is typically referred to as a *weak dual*.

Two faces (=dual vertices) are adjacent if they share at least one edge. We note that two faces may share more than one edge, which can be included in two different ways. First, we keep every edge separately making the dual graph a multigraph. Alternatively, we can condense the multiedges into ordinary ones, which requires the introduction of edge weights. We will follow the latter definition as our focus is on flow networks which are weighted anyway.

We may now define a Laplacian for the dual graph. Starting with the first definition that includes the outer face, we define, in analogy to Eq. (1), the dual Laplacian as

$$\mathbf{L}^* = \mathbf{C}^\top \mathbf{C} \in \mathbb{R}^{(L-N+2)\times(L-N+2)} . \tag{3}$$

As for the primal graph, we can decompose the Laplacian in terms of the adjacency and the degree matrix,

$$\mathbf{L}^* = \mathbf{D}^* - \mathbf{A}^*. \tag{4}$$

The dual adjacency matrix $\mathbf{A}^* \in \mathbb{R}^{(L-N+2)\times(L-N+2)}$ has the entries

$$A^*_{f,g} = \text{number of edges shared by faces } f \text{ and } g\,.$$

and the degree matrix $\mathbf{D}^* = \in \mathbb{R}^{(L-N+2)\times(L-N+2)}$ is diagonal with entries

$$D^*_{f,f} = \sum_g A_{f,g}.$$

In the second definition, we exclude the exterior face. We may again proceed to define the Graph Laplacian via the cycle incidence matrix and write

$$\mathbf{L}^*_g = \mathbf{C}^\top \mathbf{C} \in \mathbb{R}^{(L-N+1)\times(L-N+1)}. \tag{5}$$

However, this matrix is *not* a full Laplacian matrix as its row-sums are not guaranteed to vanish. Instead, $\mathbf{L}^*_g$ is a *grounded* Laplacian, that is obtained from a full Laplacian matrix by removing one row and the corresponding column [4]. Alternatively, we can define the Laplacian via the adjacency matrix as

$$\mathbf{L}^*_i = \mathbf{D}^* - \mathbf{A}^*. \tag{6}$$

The adjacency matrix $\mathbf{A}^*$ is a $(L-N+1)\times(L-N+1)$ matrix has the entries

$$A^*_{f,g} = \text{number of edges shared by faces } f \text{ and } g\,. \tag{7}$$

as before and the degree matrix $\mathbf{D}^* = \in \mathbb{R}^{(L-N+1)\times(L-N+1)}$ is diagonal with entries

$$D^*_{f,f} = \sum_g A_{f,g}.$$

Hence, the row-sums of $\mathbf{L}^*_i$ vanish as required for a Laplacian matrix. We note that the two matrices are related as

$$\mathbf{L}^*_g = \mathbf{L}^*_i + \mathbf{D}^\circ\,, \tag{8}$$

where the diagonal matrix $\mathbf{D}^\circ$ summarizes the connectivity of the interior and exterior

$$D^\circ_{f,f} = \text{number of edges shared between } f \text{ and the exterior face.} \tag{9}$$

Finally, we want to briefly comment on the two alternative definitions of the dual graph and their respective advantages and disadvantages:

- The first definition including the exterior face has two closely related advantages making this definition convenient in fundamental analysis: (i) The definitions (3) and (4) of the graph Laplacian coincide. (ii) Every edge is part of exactly two faces. This property forms the basis of MacLane's planarity criterion.

- However, the first definition is rather inconvenient in the analysis of network structures. The cycle basis is overcomplete. The additional exterior face introduces “artificial” non-local connections in the dual, which are especially unsuitable for the definition and analysis of dual communities. For community detection, we will thus work only with the adjacency matrix (7) and the Laplacian (6).
- In the analysis of flow networks we can use both definitions alike. In the first case, we have one degree of freedom that remains to be fixed. A common convention is to “ground” the exterior face – then the first definition reduces to the second one.

Weighted Graphs I

We now consider a weighted plane graph with edge weights denoted as $w_e \in \mathbb{R}_{>0}$ for all edges $e \in E$. In the analysis of graph duality and flow networks, we can include these weights in two different ways: (i) directly in the incidence matrices and (ii) via separate diagonal weight matrices. We first treat option (i) and focus on the definition of the dual that includes the exterior face.

Subsequently, we define the weighted node-edge incidence matrix $\mathbf{I} \in \mathbb{R}^{L\times N}$ as

$$I_{e,n} = \begin{cases} +\sqrt{w_e} & \text{if edge } e \text{ starts at node } n, \\ -\sqrt{w_e} & \text{if edge } e \text{ ends at node } n, \\ 0 & \text{otherwise.} \end{cases}$$

The Graph Laplacian is defined as before

$$\mathbf{L} = \mathbf{I}^\top \mathbf{I}.$$

with entries

$$L_{mn} = \begin{cases} -w_{mn} & \text{if } m \text{ is connected to } n, \\ \sum_{(m,k)\in E} w_{mk} & \text{if } m = n, \\ 0 & \text{otherwise.} \end{cases} \tag{10}$$

To ensure the defining equation for the cycle space, $\mathbf{I}^\top \mathbf{C} = 0$, the definition of the cycle edge incidence matrix must be generalized to

$$C_{\ell,f} = \begin{cases} 1/\sqrt{w_\ell} & \text{if the edge } \ell \text{ is part of face } f, \\ -1/\sqrt{w_\ell} & \text{if the reversed edge } \ell \text{ is part of face } f, \\ 0 & \text{otherwise.} \end{cases}$$

The dual Laplacian matrix is again given by the equivalent definitions (3) and (4), where the adjacency matrix is now given by

$$A^*_{f,g} = \sum_{\text{edges } e \text{ shared by faces } f \text{ and } g} \frac{1}{w_e}. \tag{11}$$

and the diagonal degree matrix is given by $D^*_{f,f} = \sum_g A_{f,g}$ as usual. We stress that the dual weights are inverse to the primal weights as expressed via Eq. (11).

Weighted Graphs II

A second option is to include the weights via an additional diagonal weight matrix

$$\mathbf{W} = \text{diag}(w_1, w_2, \dots, w_L) \in \mathbb{R}^{L\times L} \tag{12}$$

while keeping the unweighted incidence matrix $\tilde{\mathbf{I}} \in \mathbb{R}^{L\times N}$ with entries

$$\tilde{I}_{\ell,n} = \begin{cases} 1 & \text{if edge } \ell \text{ starts at node } n, \\ -1 & \text{if edge } \ell \text{ ends at node } n, \\ 0 & \text{otherwise.} \end{cases} \tag{13}$$

The Laplacian matrix of the primal graph is then defined by the definition

$$\mathbf{L} = \tilde{\mathbf{I}}^\top \mathbf{W} \tilde{\mathbf{I}}, \tag{14}$$

which coincides with Eq. (10) as desired.

We now proceed to the dual, focusing on the second definition that excludes the exterior face. The cycle edge incidence matrix $\tilde{\mathbf{C}} \in \mathbb{R}^{L\times(L-N+1)}$ now remains unweighted with components

$$\tilde{C}_{\ell,f} = \begin{cases} 1 & \text{if the edge } \ell \text{ is part of face } f, \\ -1 & \text{if the reversed edge } \ell \text{ is part of face } f, \\ 0 & \text{otherwise.} \end{cases} \tag{15}$$

The dual grounded Laplacian (5) is recovered via the definition

$$\mathbf{L}_g^* = \tilde{\mathbf{C}}^\top \mathbf{W}^{-1} \tilde{\mathbf{C}}. \tag{16}$$

Comparing to the definition of the primal Laplacian (14), we again see that dual weights are inverse to primal weights.

Supplementary Note 2: Algebraic and topological connectivity

The correspondence of algebraic and topological connectivity is well established for primal graphs. Here, we briefly review this correspondence and extend it to the dual graph.

Primal Graph

Let $G = (V, E, \mathbf{W})$ be a weighted connected graph and $\mathbf{L}$ its Laplacian. The Laplacian has one vanishing eigenvalue $\lambda_1 = 0$ with corresponding eigenvector $\vec{v}_1 \propto \vec{1} = (1, 1, \dots, 1)^\top$. Since $\mathbf{L}$ is positive semi-definite [3], we can write the eigenvalues in an ordered way

$$0 = \lambda_1 \le \lambda_2 \le \cdots \le \lambda_N. \tag{17}$$

The second eigenvalue λ_2, the Fiedler value, provides a measure of the algebraic connectivity of a graph. If it is small, we have a pronounced community structure.

We now relate the algebraic connectivity λ_2 to the topological connectivity across a cut-set of the graph. To this end, we first express λ_2 via the variational principle as

$$\lambda_2 = \min_{\vec{v} \perp \vec{1}} \frac{\vec{v}^\top \mathbf{L} \vec{v}}{\vec{v}^\top \vec{v}}\,. \tag{18}$$

We can then find an upper bound by choosing an appropriate trial vector for $\vec{v}$. To this end, we decompose the vertex set into two parts V_1 and $V_2 = V \backslash V_1$ and define a trial vector with entries

$$v_n = \begin{cases} +\left(\frac{N_2}{N\,N_1}\right)^{1/2} & \text{if } n \in V_1, \\ -\left(\frac{N_1}{N\,N_2}\right)^{1/2} & \text{if } n \in V_2, \end{cases} \tag{19}$$

where $N_1 = |V_1|$ and $N_2 = |V_2|$. Then we have

$$\begin{aligned} \lambda_2 &\le \frac{\vec{v}^\top \mathbf{L} \vec{v}}{\vec{v}^\top \vec{v}} \\ &= \frac{N_1 + N_2}{N_1\,N_2} \sum_{m \in V_1, n \in V_2} A_{m,n} \\ &= \frac{N_1 + N_2}{N_1\,N_2} \sum_{e \in S} w_e, \end{aligned} \tag{20}$$

where $S = \{(m,n) \in E | m \in V_1, n \in V_2\}$ is the cut-set corresponding to the cut $V = V_1 + V_2$.

We note that the expression (20) is not just an upper bound but gives λ_2 up to linear order in the topological connectivity. To make this more precise, assume that we start from a graph that is disconnected into two parts V_1 and $V_2 = V \backslash V_2$. We label the vertices as $V_1 = \{1, \dots, N_1\}$ and $V_2 = \{N_1 + 1, \dots, N_2 + N_2\}$. The adjacency matrix then assumes a block form

$$\mathbf{A}^{(0)} = \begin{pmatrix} \mathbf{A}_{11} & \mathbf{0} \\ \mathbf{0} & \mathbf{A}_{22} \end{pmatrix},$$

where $\mathbf{A}_1^{(0)} \in \mathbb{R}^{N_1 \times N_1}$ and $\mathbf{A}_2^{(0)} \in \mathbb{R}^{N_2 \times N_2}$. In this limit we have $\lambda_2^{(0)} = 0$. Now add some weak connections between the two modules of the graph. Then, the adjacency matrix can be written as

$$\mathbf{A} = \mathbf{A}^{(0)} + \mathbf{A}^{(1)} = \begin{pmatrix} \mathbf{A}_{11} & \mathbf{0} \\ \mathbf{0} & \mathbf{A}_{22} \end{pmatrix} + \begin{pmatrix} \mathbf{0} & \mathbf{A}_{12} \\ \mathbf{A}_{12}^\top & \mathbf{0} \end{pmatrix}$$

We now treat $\mathbf{A}^{(1)}$ as a small perturbation and apply Rayleigh-Schrödinger perturbation theory. To zeroth order we have $\lambda_2^{(0)} = 0$ and the corresponding eigenvector $\vec{v}_2^{(0)}$ coincides with the trial vector defined by Eq. (19). To linear order, the Fiedler value is then given by

$$\begin{aligned} \lambda_2 &= \lambda_2^{(0)} + \vec{v}_2^{(0)\top} \mathbf{L}^{(1)} \vec{v}_2^{(0)} + \mathcal{O}\left(\mathbf{A}_{12}^2\right) \\ &= \frac{N_1 + N_2}{N_1\,N_2} \sum_{e \in S} w_e + \mathcal{O}\left(\mathbf{A}_{12}^2\right), \end{aligned}$$

where $S = \{(m,n) \in E | m \in V_1, n \in V_2\}$ is the cut-set corresponding to the cut $V = V_1 + V_2$.

Dual Graph

We can now generalize the above treatment to the dual graph. As discussed above, we base the analysis of communities and connectivity on the dual graph that excludes the exterior face. The Laplacian of the dual graph is defined by (6) or its weighted counterpart. In particular, we have

$$\mathbf{L}_i^* = \mathbf{D}^* - \mathbf{A}^*.$$

where the entries of the adjancency matrix are given by

$$A^*_{f,g} = \sum_{\text{edges } e \text{ shared by faces } f \text{ and } g} \frac{1}{w_e}, \tag{21}$$

and $D^*_{ff} = \sum_g A^*_{fg}$.

As before, we want to define a decomposition of the graph and use the connectivity at the boundary to derive a bound for the algebraic connectivity. However, we now need the weights *along* the boundary, not *across* the boundary. To make this precise, we first define a cut-path that we will use instead of a cut-set.

Definition 1. *Let $G = (V,E)$ be a connected plane graph. The outer boundary $\mathcal{B}$ is defined as the cyclic path consisting of the edges adjacent to the exterior face and the connecting vertices.*

Definition 2. *Let $G = (V,E)$ be a connected plane graph. A cut-path is a path*

$$p = (v_1, e_1, v_2, \dots, v_{n+1}) \tag{22}$$

that includes no edges from the outer boundary $\mathcal{B}$ and that satisfies

1. *either the path is cyclic and contains at most one vertex from the boundary $\mathcal{B}$*
2. *or the path is acyclic and its start and end are on the boundary, $v_1, v_{n+1} \in \mathcal{B}$.*

We will now show that such a cut-path p indeed cuts the dual graph into two parts. Then we show that the sum of weights w_e^{-1} along the cut-path provides an upper bound for the algebraic connectivity of Fiedler value of the dual.

Lemma 1. *Let $G = (V,E)$ be connected plane graph and $G^* = (V^*, E^*)$ its dual graph. We consider a cut of the dual G^*, i.e. a decomposition of the vertex set V^* such that*

$$V_1^* \cup V_2^* = V^* \qquad \textit{and} \qquad V_1^* \cap V_2^* = \emptyset. \tag{23}$$

Then the following statements are equivalent

1. *The two dual vertex sets V_1^* and V_2^* are connected in G^**
2. *The cut set*

   $$S^* = \{e^* = (v^*, u^*) \in E^* | v^* \in V_1^*, u^* \in V_2^*\} \tag{24}$$

   is minimal.
3. *The dual of the cut set*

   $$\begin{aligned} S &= \{e \in E | e \textit{ is dual to an } e^* \in S^*\} \\ &= \{e \in E | e \textit{ is adjacent to a face } f \in V_1^* \textit{ and a face } g \in V_2^*\}. \end{aligned} \tag{25}$$

   is the edge set of a cut-path.

Proof. The equivalence of (1) and (2), i.e. the correspondence of connectedness and minimality of the cut set, is a standard result in graph theory (see, e.g., [5, Lemma 1.9.4.]).

The equivalence of (1,2) and (3) follows from the geometry of the plane embedding: A vertex $v \in V$ corresponds to a point in the plane $\mathbb{R}^2$, an edge to an arc and a face to a closed polygon. An exact proof for dual graphs that include the exterior face can be found in many text books of graph theory, see e.g. [5, Proposition 4.6.1.]. Here we briefly sketch how to extend this result to dual graphs that exclude the outer face.

(1) $\Rightarrow$ (3) Consider the embedding of the graph in the plane $\mathbb{R}^2$. Then all faces of the graph define a connected area $A \subset \mathbb{R}^2$, whose boundary ∂A corresponds to the edges in the boundary set $\mathcal{B}$. Similar, the faces in $V^*_{1,2}$ correspond to areas $A_{1,2} \subset \mathbb{R}$ such that $A_1 \cup A_2 = A$. As $V^*_{1,2}$ are connected by assumption, so are the areas $A_{1,2}$. Examples of the possible geometries are shown in Supplementary Figure 5.

The boundary between the two areas $\partial A_{1,2} = A_1 \cap A_2$ is a connected polygonal chain. It contains at most two points in the boundary ∂A of the area A, at most one if it is closed, cf. Supplementary Figure 5. Now the boundary coincides with the set

$$S = \{e \in E | e \text{ is adjacent to a face } f \in V_1^* \text{ and a face } g \in V_2^*\}.$$

which thus inherits the properties of $\partial A_{1,2}$: (i) As $\partial A_{1,2}$ is a connected polygonal chain, S is the edge set of a path. (ii) As $\partial A_{1,2}$ contains at most two points in ∂A, S contains at most two vertices from $\mathcal{B}$, at most one if the path is cyclic.

(3) $\Rightarrow$ (1) Let S be the edge set of a cut-path p. We denote this cut-path as $p = (v_1, e_1, v_2, \ldots, v_{n+1})$ and distinguish two cases. (a) If the cut-path p is acyclic, its start v_1 and end v_{n+1} are distinct and both lie on the boundary $\mathcal{B}$. We can thus write the boundary as

$$\mathcal{B} = (u_1 = v_1, \ell_1, \ldots, u_a = v_{n+1}, \ell_a, u_{a+1}, \ldots, u_{b+1} = u_1 = v_1).$$

and define two cyclic paths

$$\begin{aligned}\tilde{p}_1 &= (v_1, e_1, \ldots, v_{n+1} = u_a, \ell_a, u_{a+1}, \ldots u_{b+1} = v_1),\\ \tilde{p}_2 &= (v_1, e_1, \ldots, v_{n+1} = u_a, \ell_{a-1}, u_{a-1}, \ldots, u_1 = v_1).\end{aligned}$$

In the plane embedding, $\tilde{p}_1$ and $\tilde{p}_2$ correspond to two closed polygon chains enclosing two polygons $A_{1,2}$. As the $\tilde{p}_{1,2}$ are cyclic paths, the polygons $A_{1,2}$ are connected. We can again identify the areas $A_{1,2}$ with the dual vertex sets $V^*_{1,2}$ and conclude the following statements. (i) As $\tilde{p}_{1,2}$ are cyclic paths, the polygons $A_{1,2}$ are simply connected areas. Hence, the dual vertex sets $V^*_{1,2}$ are connected. (ii) As $\tilde{p}_{1,2}$ contain the boundary $\mathcal{B}$, we have $A = A_1 \cup A_2$ and hence $V^* = V_1^* \cup V_2^*$. (iii) As the paths $\tilde{p}_{1,2}$ do not intersect, the two polygons are disjoint, $A_1 \cap A_2 = \emptyset$, and so are the dual vertex sets, $V_1^* \cap V_2^* = \emptyset$.

(b) If the cut-path p is cyclic, its start and end coincide and lie on the boundary $v_1 = v_{n+1} \in \mathcal{B}$. In the plane embedding, the cut-path p corresponds to a closed polygon chain enclosing a polygon A_1. As p is a cyclic path, A_1 is connected. Notably, A_1 touches the boundary in exactly one point corresponding to the node $v_1 = v_{n+1}$ according to the definition of a cut-path. The area $A_2 = A \backslash A_1$ must also be connected, otherwise A_1 would touch the boundary in more than one point. We can now identify the areas $A_{1,2}$ with the dual vertex sets $V^*_{1,2}$ and conclude that both V_1^* and V_2^* are connected and satisfy $V_1^* \cap V_2^* = \emptyset$ and $V^* = V_1^* \cup V_2^*$ as desired. ☐

Corollary 1. *Let $G = (V, E, W)$ be a weighted plane graph and*

$$p = (v_1, e_1, v_2, \ldots, v_{n+1}) \tag{26}$$

be a cut-path. Then the algebraic connectivity of the dual graph G^* satisfies

$$\lambda_2^* \leq \frac{N_1^* + N_2^*}{N_1^* \, N_2^*} \sum_{e \in E(p)} w_e^{-1}, \tag{27}$$

where $E(p) = \{e_1, \ldots, e_n\}$ is the set of edges in the cut-path and $N^*_{1,2} = |V^*_{1,2}|$

Proof. In full analogy to the analysis of the primal graph (cf. (20)), we find the inequality

$$\lambda_2^* \leq \frac{N_1^* + N_2^*}{N_1^* \, N_2^*} \sum_{c \in V_1^*, d \in V_2^*} A^*_{c,d},$$

where V_1^* and $V_2^* = V^* \backslash V_2^*$is a decomposition of the vertex set of the dual and $N_{1,2}^* = |V_{1,2}^*|$. The entries of the dual adjacency matrix are given by Eq. (21) such that we have

$$\begin{aligned} \lambda_2^* &\leq \frac{N_1^* + N_2^*}{N_1^* N_2^*} \sum_{c \in V_1^*, d \in V_2^*} \sum_{\text{edges } e \text{ shared by faces } c \text{ and } d} w_e^{-1} \\ &= \frac{N_1^* + N_2^*}{N_1^* N_2^*} \sum_{e \in S} w_e^{-1} \end{aligned}$$

where the last equality follows from Lemma 1. □

Supplementary Note 3: Linear flow networks

Linear flow networks describe a generic model for many types of supply networks including AC power grids [6–8], DC electric circuits [9–11], hydraulic networks [12, 13], and vascular networks of plants [14]. Assume that the underlying network is given by a graph $G = (E, V)$ with $N = |V|$ nodes and $M = |E|$ edges. Then we assign a potential $\theta_n \in \mathbb{R}$ to each node $n \in V$. This potential has the following interpretation: In AC power grids in the linear power flow approximation, it denotes the voltage phase angle whereas in DC electric circuits, it refers to the voltage level at node n. Finally, in hydraulic or vascular networks, θ_n denotes the pressure at node n. Then the flow F_ℓ along a link $\ell = (m, n)$ is given by

$$F_\ell = w_\ell(\theta_m - \theta_n), \tag{28}$$

where w_ℓ is the weight of the link ℓ. In AC power grids in the linear power flow approximation, w_ℓ is proportional to the link's susceptance, in resistor networks it is given by the link's conductance and in a hydraulic or vascular network it depends on the geometry of the pipe or vein. Note that all these networks are undirected, i.e. flow is possible in both directions alike, such that $w_{m,n} = w_{n,m}$. Since the flow (28) depends only on the potential drop, the potentials θ_n are only determined up to a constant phase shift applied to all nodes. This problem may be solved by assigning a reference potential to a preselected slack node n, e.g. setting $\theta_n := 0$.

Now assume that there is an inflow P_m at every node $m \in V$. The value of P_m is positive if a current, power, or fluid is injected to the node and negative if it is withdrawn from the node. Here and in the following, we assume that the in- and outflows sum to zero, $\sum_i^N P_i = 0$, and call them 'balanced'. The flows F_ℓ along the edges $\ell \in E$ may then be determined by using the continuity equation, also referred to as Kirchhoff's current law (KCL)

$$\sum_{\ell \in E} \tilde{I}_{\ell,n} F_\ell = P_n, \qquad \forall\, n \in V. \tag{29}$$

Here, $\tilde{I}_{\ell,n}$ are the entries of the graph's unweighted edge-node incidence matrix (13). The potentials θ_n which fulfil the continuity equation (29) and the equation for the flows (28) automatically satisfy Kirchhoff's voltage law which states that the potential drop around any closed loop $\mathcal{C}$ needs to vanish

$$\sum_{(n,m) \in \mathcal{C}} \theta_n - \theta_m = 0. \tag{30}$$

Defining a vector of flows $\vec{F} = (F_1, .., F_M)^\top \in \mathbb{R}^M$, a vector of potentials $\vec{\theta} = (\theta_1, ..., \theta_N)^\top \in \mathbb{R}^N$ and a vector of inflows $\vec{P} = (P_1, ..., P_N)^\top \in \mathbb{R}^N$, we can write these relationships in a more compact form. Kirchhoff's current law (29) in vectorized form reads as

$$\tilde{\mathbf{I}}^\top \vec{F} = \vec{P}.$$

In addition to that, we can write the relationship between flows and potentials in vectorized form,

$$\vec{F} = \mathbf{W} \tilde{\mathbf{I}} \vec{\theta}. \tag{31}$$

Here, $\mathbf{W} = \mathrm{diag}(w_1, ..., w_L) \in \mathbb{R}^{M \times M}$ is a diagonal matrix summarizing the edge weights, cf. Eq. 12. Now we can put together the last two equations to arrive at a discrete Poisson equation for the nodal potentials

$$\mathbf{L}\vec{\theta} = \vec{P}. \tag{32}$$

Here, $\mathbf{L} = \tilde{\mathbf{I}}^\top \mathbf{W} \tilde{\mathbf{I}}$ is the Laplacian matrix.

Quantifying the impact of perturbations - primal graph

We now use the notation developed in the last section to study the effect of perturbations, in particular links failures, and derive the expression for the sensitivity factor $\eta_{i,k,\ell}$ given in the main text. Assume that the inflow at one node i is increased by an amount ΔP and decreased at another node k by the same amount. The inflow vector changes as $\vec{P} \to \vec{P}' = \vec{P} + \Delta\vec{\theta}$ with

$$\Delta\vec{P} = \Delta P(\vec{e}_i - \vec{e}_k),$$

where $\vec{e}_i$ denotes the ith standard unit vector. As a consequence, the nodal potentials changes as $\vec{\theta} \to \vec{\theta}' = \vec{\theta} + \Delta\vec{\theta}$. Both the new and the old potentials have to fulfil the Poisson equation (32), Subtracting the two equations, we arrive at an explicit equation for the change in nodal potentials

$$\mathbf{L}\Delta\vec{\theta} = \Delta P(\vec{e}_i - \vec{e}_k).$$

Note that the Laplacian matrix of a connected graph always has one zero eigenvalue [3]. Thus, we cannot simply invert this equation to calculate the change in the nodal potentials $\Delta\vec{\theta}$. This problem is typically solved by making use of the Moore-Penrose pseudoinverse of the Laplacian matrix $\mathbf{L}^\dagger$ which has properties similar to the matrix inverse [15].

Now we can use Kirchhoff's current law (29) to calculate the resulting changes in the flows

$$\Delta\vec{F} = \mathbf{W}\tilde{\mathbf{I}}\mathbf{L}^\dagger(\vec{e}_i - \vec{e}_k)\Delta P.$$

Finally, we can read off the change of the flow ΔF_ℓ on a line ℓ. We define an indicator vector $\vec{l}_\ell$ as the ℓth standard unit vector in $\vec{R}^M$ and obtain

$$\Delta F_\ell = \vec{l}_\ell^\top \Delta\vec{F} = w_\ell \vec{l}_\ell^\top \tilde{\mathbf{I}}\mathbf{L}^\dagger(\vec{e}_i - \vec{e}_k)\Delta P = \sqrt{w_\ell}\vec{l}_\ell^\top \mathbf{I}\mathbf{L}^\dagger(\vec{e}_i - \vec{e}_k)\Delta P, \tag{33}$$

where we replaced the unweighted incidence matrix $\tilde{\mathbf{I}}$ by the weighted matrix $\mathbf{I}$. Then the sensitivity factor reads

$$\eta_{i,k,\ell} = \frac{\Delta F_\ell}{\Delta P} = \sqrt{w_\ell}\vec{l}_\ell^\top \mathbf{I}\mathbf{L}^\dagger(\vec{e}_i - \vec{e}_k). \tag{34}$$

Note that the sensitivity factor is purely topological, i.e. it may be calculated only based on the network topology and weights and does *not* depend on the in- and outflows. If the two nodes i and k are the terminal nodes of an edge $p = (i, k)$, we can identify

$$\vec{e}_i - \vec{e}_k = \tilde{\mathbf{I}}^\top \vec{l}_p,$$

and the sensitivity factor reads

$$\eta_{p,\ell} = w_\ell \vec{l}_\ell^\top \tilde{\mathbf{I}}\mathbf{L}^\dagger \tilde{\mathbf{I}}^\top \vec{l}_p = \sqrt{w_\ell / w_p}\vec{l}_\ell^\top \mathbf{I}\mathbf{L}^\dagger \mathbf{I}^\top \vec{l}_p.$$

Quantifying the impact of perturbations - dual graph

The discussion of perturbations in the in- and outflows developed in the last section was recently extended to the dual graph [16, 17]. We will briefly cover this here and derive the dual expression for the sensitivity factor η. As before, we assume that the inflow at one node i is increased by an amount ΔP and decreased at another node k by the same amount such $\Delta\vec{P} = \Delta P(\vec{e}_i - \vec{e}_k)$. By subtracting Kirchhoff's current law (29) for the situation before and after the change in inflow, we arrive at

$$\tilde{\mathbf{I}}^\top \Delta\vec{F} = \Delta P(\vec{e}_i - \vec{e}_k),$$

which leaves us with an underdetermined system of equations: We have M unknown flow changes ΔF_ℓ, but only $N - 1$ equations as one is redundant. The general solution can thus be written as

$$\Delta\vec{F} = \Delta\vec{F}_{\text{part}} + \Delta\vec{F}_{\text{hom}}\,, \tag{35}$$

where $\Delta\vec{F}_{\text{part}}$ is one particular solution of the equation and $\Delta\vec{F}_{\text{hom}}$ is an arbitrary solution of the homogeneous equation, i.e. an arbitrary solution of $\tilde{\mathbf{I}}^\top \Delta\vec{F}_{\text{hom}} = \vec{0}$. We can now use graph duality to parametrize the homogeneous solutions and to single out the physically correct solution.

The kernel of the node-edge incidence matrix is spanned by the cycle-edge incidence matrix

$$\tilde{\mathbf{I}}^\top \tilde{\mathbf{C}} = 0,$$

where we here use the unweighted matrix (15). Hence, we can write the homogeneous solutions as

$$\Delta\vec{F}_{\text{hom}} = \tilde{\mathbf{C}}\vec{f},$$

where $\vec{f} \in \mathbb{R}^{M-N+1}$ is a vector of cycle flows – one for each cycle. The physically correct values of the cycle flows are then determined by Kirchhoff's voltage law (Eq. 30) which may be written compactly as

$$\tilde{\mathbf{C}}^\top \mathbf{W}^{-1} \Delta \vec{F} = 0, \tag{36}$$

where we used that $\Delta\theta_n - \Delta\theta_m = \Delta F_{nm}/w_{nm}$. Inserting equations (35) and (36), we then obtain

$$\begin{aligned} \tilde{\mathbf{C}}^\top \mathbf{W}^{-1} \tilde{\mathbf{C}} \vec{f} &= -\tilde{\mathbf{C}}^\top \mathbf{W}^{-1} \Delta \vec{F}_{\text{part}}, \\ \Leftrightarrow \quad \mathbf{L}_g^* \vec{f} &= -\tilde{\mathbf{C}}^\top \mathbf{W}^{-1} \Delta \vec{F}_{\text{part}}, \end{aligned} \tag{37}$$

with the dual Laplacian $\mathbf{L}_g^* = \tilde{\mathbf{C}}^\top \mathbf{W}^{-1} \tilde{\mathbf{C}}$. We thus found a discrete Poisson equation for the cycle flows $\vec{f}$ with the grounded dual Laplacian $\mathbf{L}_g^*$ in direct correspondence to its primal counterpart (32). We can now proceed as for the primal graph: Inverting the discrete Poisson equation for the cycle flows and plugging the result into Eq. (35), we thus arrive at

$$\Delta \vec{F} = (\mathbf{1}_M - \tilde{\mathbf{C}} (\mathbf{L}_g^*)^{-1} \tilde{\mathbf{C}}^\top \mathbf{W}^{-1}) \Delta \vec{F}_{\text{part}}.$$

Here, $\mathbf{1}_M$ denotes the identity matrix of dimensions $M \times M$. As a last step, we need to determine a particular solution $\Delta \vec{F}_{\text{part}}$. This can be accomplished as follows [17]: Let $\mathcal{T}^{ik} \in \mathbb{R}^M$ be a vector determining an (arbitrary) path between the node i with inflow ΔP and node k with the same outflow. The entries of $\mathcal{T}^{ik}$ are given as follows

$$\mathcal{T}_e^{ik} = \begin{cases} 1 & \text{if edge } e \text{ is element of the path } i \to k, \\ -1 & \text{if reversed edge } e \text{ is element of the path } i \to k, \\ 0 & \text{otherwise.} \end{cases}$$

Then a particular solution is given by $\Delta \vec{F}_{\text{part}} = \mathcal{T}^{ik} \Delta P$, and we can plug this into the above equation

$$\begin{aligned} \Delta \vec{F} &= (\mathbf{1}_M - \tilde{\mathbf{C}} (\mathbf{L}_g^*)^\dagger \tilde{\mathbf{C}}^\top \mathbf{W}^{-1}) \mathcal{T}^{ik} \Delta P, \\ \Rightarrow \Delta F_\ell = \vec{l}_\ell^\top \Delta \vec{F} &= \left(\vec{l}_\ell^\top \mathcal{T}^{ik} - \vec{l}_\ell^\top \tilde{\mathbf{C}} (\mathbf{L}_g^*)^\dagger \tilde{\mathbf{C}}^\top \mathbf{W}^{-1} \mathcal{T}^{ik} \right) \Delta P. \end{aligned}$$

If nodes i and k are the two terminal ends of an edge $e = (i,k) \neq \ell$, the path vector may be identified with the edge's indicator vector, $\mathcal{T}^{ik} = \vec{l}_e$. Then we can simplify the above expression for any edge $\ell \neq e$ to

$$\Delta F_\ell = -w_p^{-1} \vec{l}_\ell^\top \tilde{\mathbf{C}} (\mathbf{L}_g^*)^\dagger \tilde{\mathbf{C}}^\top \vec{l}_e \Delta P,$$

Notably, the first expression in Eq. (38) vanishes since $\vec{l}_\ell^\top \vec{l}_e = \delta_{\ell e}$. Thus, we can identify the sensitivity factor $\eta_{i,k,\ell}$ as

$$\eta_{i,k,\ell} = -w_e^{-1} \vec{l}_\ell^\top \mathbf{C} (\mathbf{L}_g^*)^\dagger \mathbf{C}^\top \vec{l}_e = -\sqrt{\frac{w_\ell}{w_e}} \vec{l}_\ell^\top \mathbf{C} (\mathbf{L}_G^*)^\dagger \mathbf{C}^\top \vec{l}_e \,.$$

Modelling link failures

The sensitivity factor $\eta_{i,k,\ell}$ may also be used to describe the failure of links. Assume that a link e fails, thus loosing its ability to carry flow and setting the weight to zero, $w_e = 0$. Instead of removing the link from the network, we can find an analogous description based on the sensitivity factor [6, 18].

Assume that the link $e = (e_1, e_2)$ carries a flow F_e before the outage. Now we model the removal of the link by an in- and outflow at the two ends of the link: To this end, we assume that the line was disconnected from the network and consider a fictitious flow $\hat{F}_e$ which is the result of a (fictitious) inflow $\Delta P = \hat{F}_e$ at the starting node e_1 and an outflow of the same amount at the terminal node e_2 of link e. On the other hand, we can also calculate the flow $\hat{F}_e$ flowing on line e after the injection by a self-consistency argument: It can be calculated using the sensitivity factor

$$\begin{aligned} \hat{F}_e &= F_e + \eta_{e_1,e_2,e} \Delta P = F_e + \eta_{e_1,e_2,e} \hat{F}_e \\ \Rightarrow \Delta P &= \hat{F}_e = F_e (1 - \eta_{e_1,e_2,e})^{-1}. \end{aligned}$$

Now we can calculate the change in the flow on another link ℓ due to the failure of link e as

$$\Delta F_\ell = \eta_{e_1,e_2,\ell} \Delta P = \frac{\eta_{e_1,e_2,\ell}}{1 - \eta_{e_1,e_2,e}} F_e.$$

We can thus calculate the flow change ΔF_ℓ on any link ℓ due to the failure of another link e based on the initial flow on link e and the sensitivity factor η. The expression $\eta_{e_1,e_2,\ell}(1-\eta_{e_1,e_2,e})^{-1}$ is known as *Line Outage Distribution Factor* in power system security analysis [6]. Thus, both changes in the inflows and link failures can be captured by the sensitivity factor η.

To quantify the effect of (dual) communities on network robustness in linear flow networks we defined the ratio of flow changes in the main text (cf. Ref. [19]),

$$R(\ell,d) = \frac{\langle|\Delta F_k|\rangle_d^{k\in \mathrm{O}}}{\langle|\Delta F_r|\rangle_d^{r\in \mathrm{S}}}.$$

Importantly, the ratio can be used to quantify both, the effect of communities on changes in the inflow patterns and on failure spreading. For an inflow and outflow of ΔP at the two terminal ends of the link e, e_1 and e_2, respectively, the ratio is calculated as

$$R(e,d) = \frac{\langle|\Delta F_k|\rangle_d^{k\in \mathrm{O}}}{\langle|\Delta F_r|\rangle_d^{r\in \mathrm{S}}} = \frac{\langle|\eta_{e_1,e_2,k}\Delta P|\rangle_d^{k\in \mathrm{O}}}{\langle|\eta_{e_1,e_2,r}\Delta P|\rangle_d^{r\in \mathrm{S}}} = \frac{\langle|\eta_{e_1,e_2,k}|\rangle_d^{k\in \mathrm{O}}}{\langle|\eta_{e_1,e_2,r}|\rangle_d^{r\in \mathrm{S}}}.$$

On the other hand, if we instead calculate the ratio for the failure of a link e with initial flow F_e, we arrive at

$$R(e,d) = \frac{\langle|\Delta F_k|\rangle_d^{k\in \mathrm{O}}}{\langle|\Delta F_r|\rangle_d^{r\in \mathrm{S}}} = \frac{\langle|\eta_{e_1,e_2,k}(1-\eta_{e_1,e_2,e})^{-1}F_e|\rangle_d^{k\in \mathrm{O}}}{\langle|\eta_{e_1,e_2,r}(1-\eta_{e_1,e_2,e})^{-1}F_e|\rangle_d^{r\in \mathrm{S}}} = \frac{\langle|\eta_{e_1,e_2,k}|\rangle_d^{k\in \mathrm{O}}}{\langle|\eta_{e_1,e_2,r}|\rangle_d^{r\in \mathrm{S}}}. \tag{38}$$

Thus, in both cases, the ratio is determined by the sensitivity factor η which is in turn governed by the Pseudo-inverse of the Laplacian matrix $\mathbf{L}^\dagger$ or the Pseudo-inverse of the dual Laplacian $(\mathbf{L}_g^*)^\dagger$ when formulating the problem in the dual graph.

Connectivity structure determines network response to link failures

In this section, we will demonstrate why a weak connection between two components of a network limits the flow changes in one component when a link in the other one fails. Our analysis follows the approach towards perturbation spreading used in Manik et al. [20, 21] that is based on Rayleigh-Schrödinger perturbation theory [22].

Consider a connected graph $G=(E,V)$ with N nodes and M edges consisting of two subgraphs $G_1=(E_1,V_1)$ and $G_2=(E_2,V_2)$ with n_1 nodes and $n_2=N-n_1$ nodes, respectively, that are mutually weakly connected. Here, a weak connection between the two subgraphs may either be realized through a (relatively) weak number of links in case of an unweighted graph or the overall weight of the connections between the two subgraphs being small [5]. We then sort the graph's vertices V in such a way that the first n_1 vertices belong to the first subgraph G_1 and the other n_2 vertices belong to the second one G_2. We may regard the weak connections between the to subgraphs as a perturbation to the graph in which the two subgraphs are disconnected. In terms of the graph Laplacian $\mathbf{L}$, we thus write

$$\mathbf{L} = \mathbf{L}_0 + \tilde{\mathbf{L}} = \begin{pmatrix} \mathbf{L}_1 & \mathbf{0}_{n_1\times n_2} \\ \mathbf{0}_{n_2\times n_1} & \mathbf{L}_2 \end{pmatrix} + \begin{pmatrix} \mathbf{D}_{12} & -\mathbf{A}_{12} \\ -\mathbf{A}_{12}^\top & \mathbf{D}_{21} \end{pmatrix}.$$

Here, $\mathbf{L}_0$ is the graph Laplacian for the graph when disconnecting the two subgraphs, expressed in terms of their Laplacian matrices $\mathbf{L}_1 \in \mathbb{R}^{n_1\times n_1}$ and $\mathbf{L}_2 \in \mathbb{R}^{n_2\times n_2}$ and $\tilde{\mathbf{L}}$ is the perturbation matrix with the diagonal degree matrices $\mathbf{D}_{12}$ and $\mathbf{D}_{21}$ denoting the degree for the graph connecting the two subgraphs. $\mathbf{A}_{12}$ is the adjacency matrix for this graph that is assumed to be relatively sparse, thus indicating the weak inter-subgraph connections.

To examine the effect of link failures, we need to study the pseudoinverse of this matrix. Therefore, we denote by $\mathbf{X}=\mathbf{L}^\dagger$ the Moore-Penrose pseudoinverse of the overall graph Laplacian and by $\mathbf{X}_1=\mathbf{L}_1^\dagger$ and $\mathbf{X}_2=\mathbf{L}_2^\dagger$ the Moore-Penrose pseudoinverses of the subgraphs' Laplacian matrices $\mathbf{L}_1$ and $\mathbf{L}_2$, respectively. For the inverse $\mathbf{X}_0$ of the unperturbed Laplacian $\mathbf{L}_0$, we then get

$$\mathbf{X}_0 = \begin{pmatrix} \mathbf{X}_1 & \mathbf{0}_{n_1\times n_2} \\ \mathbf{0}_{n_2\times n_1} & \mathbf{X}_2 \end{pmatrix}.$$

In order to calculate the matrix inverse $\mathbf{X}$, we can expand this matrix using the Neumann series (see e.g. Ref. [23]). We note that some issues may arise in this perturbative treatment and the series expansion, as the Moore-Penrose

pseudo-inverse is not continuous in general. However, these this does not apply here, since we can easily circumvent the formal problems by fixing the phase of an arbitrarily chosen slack node n as $\theta_n \equiv 0$ and removing the n-th row from the linear set of equations. The resulting set of equations is of full rank and admits a unique solution. The corresponding matrices are again grounded Laplacians [4] which are invertible. The matrix inverse is continuous and admits a series expansion as in Equation (39). For the sake of notational simplicity and coherence, we will however stick to the ordinary Laplacians. The matrix inverse $\mathbf{X}$ then reads

$$\begin{aligned}\mathbf{X} = (\mathbf{L}_0 + \tilde{\mathbf{L}})^\dagger &= \left[\mathbf{L}_0\left(\mathbf{1} + \mathbf{X}_0\tilde{\mathbf{L}}\right)\right]^\dagger = \left[\left(\mathbf{1} + \tilde{\mathbf{L}}\mathbf{X}_0\right)\mathbf{L}_0\right]^\dagger \\ &= \mathbf{X}_0\left[\left(\mathbf{1} - (-\mathbf{X}_0\tilde{\mathbf{L}})\right)\right]^\dagger = \mathbf{X}_0\sum_{k=1}^{\infty}(-1)^k(\tilde{\mathbf{L}}\mathbf{X}_0)^k,\end{aligned}$$

where we inserted the Neumann series in the last step. We can thus approximate the matrix inverse of the graph Laplacian as

$$\mathbf{X} = \mathbf{X}_0 - \mathbf{X}_0\tilde{\mathbf{L}}\mathbf{X}_0 + \mathcal{O}(\tilde{\mathbf{L}}^2),$$

where $\mathcal{O}(\tilde{\mathbf{L}}^2)$ denotes terms of at least order two in the perturbation matrix $\tilde{\mathbf{L}}$.

Now we can use these expressions to calculate a first order approximation for the sensitivity factor $\eta_{i,k,\ell}$ in the weakly connected limit. Assume we are monitoring the flow changes on line ℓ with indicator vector $\vec{l_\ell}$ and define $\vec{\nu}_\ell = \mathbf{I}\cdot\vec{l_\ell} = \vec{e}_{\ell_1} - \vec{e}_{\ell_2}$ as a result of a power transfer along line $k = (k_1, k_2)$ with indicator vector $\vec{l_k}$ and $\vec{\nu}_k = \mathbf{I}\cdot\vec{l_k}$. We can then calculate the sensitivity factor as

$$\eta_{k_1,k_2,\ell} = w_\ell\vec{\nu}_\ell^\top\mathbf{X}\vec{\nu}_k.$$

Now we distinguish two cases. First assume that ℓ and k are contained in the same subgraph, say G_1. In this case, we can write with slight abuse of notation $\vec{\nu}_\ell = (\hat{\vec{\nu}}_\ell, \vec{0}_{n_2})^\top$ and $\vec{\nu}_k = (\hat{\vec{\nu}}_k, \vec{0}_{n_2})^\top$, where $\hat{\vec{\nu}}_l \in \mathbb{R}^{n_1}$ denotes the projection of the vector onto the subspace of vertices the first subgraph G_1 and the whole vector $\vec{\nu}_\ell$ is still understood as a vector in $\mathbb{R}^N$. In this case, we may write the sensitivity factor as

$$\eta_{k_1,k_2,\ell} = w_\ell(\hat{\vec{\nu}}_\ell^\top, \vec{0}_{n_2}^\top)\mathbf{X}\begin{pmatrix}\hat{\vec{\nu}}_k\\ \vec{0}_{n_2}\end{pmatrix} = w_\ell\hat{\vec{\nu}}_\ell^\top\mathbf{X}_1\hat{\vec{\nu}}_k + \mathcal{O}(\tilde{\mathbf{L}}). \tag{39}$$

Now consider the case where ℓ and k are contained in different modules of the network. In this case, we may write $\vec{\nu}_\ell = (\hat{\vec{\nu}}_\ell, \vec{0}_{n_2})^\top$ and $\vec{\nu}_k = (\vec{0}_{n_1}, \hat{\vec{\nu}}_k)^\top$ and calculate the sensitivity factor as

$$\begin{aligned}\eta_{k_1,k_2,\ell} &= w_\ell(\hat{\vec{\nu}}_\ell^\top, \vec{0}_{n_2}^\top)\mathbf{X}\begin{pmatrix}\vec{0}_{n_1}\\ \hat{\vec{\nu}}_k\end{pmatrix} \\ &= w_\ell(\hat{\vec{\nu}}_\ell^\top, \vec{0}_{n_2}^\top)\left[\mathbf{X}_0 + \mathbf{X}_0\tilde{\mathbf{L}}\mathbf{X}_0\right]\begin{pmatrix}\vec{0}_{n_1}\\ \hat{\vec{\nu}}_k\end{pmatrix} + \mathcal{O}(\tilde{\mathbf{L}}^2) \\ &= w_\ell\left[0 + (\hat{\vec{\nu}}_\ell^\top\mathbf{X}_1, \vec{0}_{n_2}^\top)\tilde{\mathbf{L}}\begin{pmatrix}\vec{0}_{n_1}\\ \mathbf{X}_2\hat{\vec{\nu}}_k\end{pmatrix}\right] + \mathcal{O}(\tilde{\mathbf{L}}^2).\end{aligned}$$

We thus observe that the leading order contribution of the perturbation matrix to the sensitivity factor is $\tilde{\mathbf{L}}^0$ if both links are contained in the same module and $\tilde{\mathbf{L}}^1$ if they are contained in different modules.

Now we compare this to the scaling of Laplacian eigenvalues with the perturbation matrix. The first eigenvector of $\mathbf{L}_0$ is given by the constant shift $\vec{v}_1 = N^{-1/2}\vec{1}_N$ and has eigenalue $\lambda_1 = 0$. In the weakly connected limit considering only $\mathbf{L}_0$, the Fiedler eigenvalue vanishes as well $\lambda_2^{(0)} = 0$, and has an associated eigenvector [20]

$$\vec{v}_2^{(0)} = N^{-1/2}(\underbrace{\sqrt{n_2/n_1}, ..., \sqrt{n_2/n_1}}_{n_1\text{ times}}, \underbrace{-\sqrt{n_1/n_2}, ..., -\sqrt{n_1/n_2}}_{n_2\text{ times}})^\top.$$

Here, we use the superscript (0) to denote the eigenvector and eigenvalue in the unperturbed case. A first order estimate for the Fiedler value may thus be calculated by using Rayleigh Schrödinger perturbation theory as

$$\lambda_2^{(1)} = (\vec{v}_2^{(0)})^\top\tilde{\mathbf{L}}\vec{v}_2^{(0)} + \mathcal{O}(\tilde{\mathbf{L}^2}). \tag{40}$$

For weakly connected graphs, we thus expect the sensitivity factor $\eta_{\ell_1,\ell_2,k}$ to scale with the connectivity of the graph in the same way as the Fiedler value of the graph if ℓ and k lie in different communities due to the fact that $\lambda_2^{(1)} \propto \tilde{\mathbf{L}}$ and expect it to be to leading order independent of the Fiedler value if ℓ and k are in the same community.

Flow ratio scales with increasing connectivity between weakly connected modules

In the main part of the manuscript, we consider the ratio $R(\ell, d)$ between the flow changes $\Delta F_{i\to j}$ within the other community $(i,j) \in \mathrm{O}$ and the flow changes in the same community $(i,j) \in \mathrm{S}$ after a link failure. It is well established that the flow changes typically decay with distance to the failing link [18, 24–26]. To be able to neglect this effect on the flow changes, we take the average absolute flow changes at a fixed distance d denoted by $\langle |\Delta F_{i\to j}|\rangle_d^{(i,j)\in \mathrm{S}}$ for the same community and $\langle |\Delta F_{i\to j}|\rangle_d^{(i,j)\in \mathrm{O}}$ for the other community where we average over all edges $\ell = (i,j)$ within the respective community that are located at an unweighted edge distance of d to the failing link. With this formalism at hand, we can now estimate the scaling of this ratio with the strength of the perturbation. Suppose that link $k = (r,s)$ is failing. Then the flow ratio reads (cf. Eq. (38))

$$R(\ell, d) = \frac{\langle |\eta_{\ell_1,\ell_2,k}|\rangle_d^{k\in \mathrm{O}}}{\langle |\eta_{\ell_1,\ell_2,r}|\rangle_d^{r\in \mathrm{S}}}.$$

Now we can make use of our results on the scaling of the sensitivity factors with increasing connectivity between the subnetworks. From Eqs. (39) and, (40) we see that this ratio scales with the first order of the perturbation

$$\begin{aligned} R(\ell, d) &= \frac{\left\langle \left| w_\ell (\hat{\vec{\nu}}_\ell^\top \mathbf{X}_1, \vec{0}_{n_2}^\top)\tilde{\mathbf{L}} \begin{pmatrix} \vec{0}_{n_1} \\ \mathbf{X}_2 \hat{\vec{\nu}}_k \end{pmatrix} \right| \right\rangle_d^{k=(i,j)\in \mathrm{O}} + \mathcal{O}(\tilde{\mathbf{L}}^2)}{\left\langle \left| w_\ell \hat{\vec{\nu}}_\ell^\top \mathbf{X}_1 \hat{\vec{\nu}}_r \right| \right\rangle_d^{r=(p,q)\in \mathrm{S}} + \mathcal{O}(\tilde{\mathbf{L}})} \\ \Rightarrow & R \propto \tilde{\mathbf{L}} + \mathcal{O}(\tilde{\mathbf{L}}^2) \end{aligned}$$

in the weakly connected limit assuming without loss of generality that the failing link is located in the first subgraph G_1, $\ell \in E(G_1)$. Since we deduced in Eq. (40) that the Fiedler value scales with the perturbation in the first order as well, we expect the two quantities to show a similar scaling with the perturbation matrix $\tilde{\mathbf{L}}$.

Note that the derivation works exactly the same way for the dual graph G^* and dual Fiedler value λ_2^*: We simply have to replace the sensitivity factor $\eta_{\ell_1,\ell_2,k}$ by its dual representation.

Supplementary Note 4: Fluctuating sink model with additive Dirichlet noise

We start by a review of the original single-sink model as formulated by Corson [1]. Consider a linear flow network on a graph G with N nodes and M edges summarized in the node set V and edge set E. Then we choose one vertex to be the source of the network while all the others are sinks. We assume that the outflow at the sinks fluctuates in time, which is modelled by uncorrelated, iid Gaussian variables $P_i \sim \mathcal{N}(\mu, \sigma),\ i \in \{2, ..., N\}$. Sources and sinks have to balance at any point in time such that

$$\sum_{i=1}^{N} P_i = 0. \tag{41}$$

This equation immediately yields the statistical properties of the source as a consequence of the statistics of the sinks. We label the nodes such that the source has the index $i = 1$ and obtain

$$\langle P_1 \rangle = -(N-1)\mu,$$

where $\langle \cdot \rangle$ denotes the mean over different realizations of the Gaussian variables.

The network structure described by the edge weights w_ℓ is then optimized to minimize the average dissipation

$$\langle D \rangle = \sum_{\ell=1}^{M} \frac{\langle F_\ell^2 \rangle}{w_\ell}.$$

Here, $\langle F_\ell^2 \rangle$ is the second moment of the flows which are determined by the statistics of the sources and sinks by virtue of Kirchhoff's equations. However, we assume that the budget for constructing or strengthening edges is limited leading to a resource constraint

$$\sum_{\ell \in E} w_\ell^\gamma \leq 1.$$

Here, γ is a cost parameter that controls how expensive it is to increase the weight of an edge. The main challenge when performing the minimization is the interdependence between link weights and flows which cannot be varied independently. Solving the constrained optimization problem with using the method of Lagrange multipliers yields two conditions: First, the flows are determined by the edge weights w_ℓ and the inflows P_i via Kirchhoff's equations. Second, the optimal weights are related to the flows as

$$w_\ell = \frac{\langle F_\ell^2 \rangle^{1/(1+\gamma)}}{\left(\sum_{e \in E} \langle F_e^2 \rangle^{\gamma/(1+\gamma)}\right)^{1/\gamma}}. \tag{42}$$

Corson [1] and Katifori et al. [14] independently came up with a procedure to solve this problem in an iterative self-consistent manor starting from a random initial guess for the edge weights. Then one out the following steps repeatedly until the procedure converges:

1. The moments of the flows are determined via Kirchhoff's equations.

2. The weights are updated according to Eq. (42).

Notably, the system can admit several local minima, and it depends on the initial guess, to which minimum the iteration converges.

Now we extend this set-up to a network with multiple sources. Assume that we again label the nodes such that the first N_s nodes are sources and the remaining $N - N_s$ nodes are sinks, which are still uncorrelated, iid Gaussian variables. However, when considering more than one source, $N_s > 1$, the distribution of the sources is not completely determined by Equation (41), but has additional degrees of freedom. We use this degree of freedom to put additive Dirichlet noise $X_i \sim \mathrm{Dir}(\alpha)$ on the sources. In particular, we assume that

$$P_i = -\frac{1}{N_s} \sum_{i=N_s+1}^{N} P_i + K \left(\frac{1}{N_s} - X_i \right), \quad X_i \sim \mathrm{Dir}(\alpha), i = 1, \ldots, N_s,$$

where $K \in \mathbb{R}$ is a scaling parameter. Importantly, the Dirichlet variables sum to unity for all realizations,

$$\sum_{i=1}^{N_s} X_i = 1. \tag{43}$$

The parameter α determines the shape of the dirichlet distribution and tunes the variability of the sources. For $\alpha = 1$, the distribution is flat such that the individual X_i fluctuate strongly. For $\alpha \gg 1$, the distribution strongly peaks around the mean such that fluctuations of the individual X_i are small.

The moments of the Dirichlet random variables are given by

$$\begin{aligned}
\langle X_i \rangle &= \frac{1}{N_s} \\
\langle X_i^2 \rangle &= \frac{(N_s - 1)}{N_s^2(N_s\alpha + 1)} + \frac{1}{N_s^2} \\
\langle X_i X_j \rangle &= -\frac{1}{N_s^2(N_s\alpha + 1)} + \frac{1}{N_s^2}.
\end{aligned}$$

Note that this results in a vanishing sum over the second moments

$$\sum_{i=1}^{N_s} \langle X_i X_j \rangle = 0,$$

by virtue of the definition of the Dirichlet parameter and the first equation. Now we can shift to new random variables $Y_i = K\left(\frac{1}{N_s} - X_i\right)$ with zero mean, $\langle Y_i \rangle = 0$, and the following second moments

$$\begin{aligned}
\langle Y_i Y_j \rangle &= K^2 \left\langle \left(\frac{1}{N_s} - X_i\right)\left(\frac{1}{N_s} - X_j\right)\right\rangle \\
&= K^2 \left(-\frac{1}{N_s^2} + \langle X_i X_j \rangle\right) \\
&= -K^2 \frac{1}{N_s^2(N_s\alpha + 1)}, \quad i \neq j, \\
\langle Y_i^2 \rangle &= K^2 \left\langle \left(\frac{1}{N_s} - X_i\right)^2 \right\rangle = K^2 \frac{(N_s - 1)}{N_s^2(N_s\alpha + 1)}
\end{aligned}$$

such that the sum over the second moment still vanishes $\sum_{i=1}^{N_s} \langle Y_i Y_j \rangle = 0$.

With these results we can compute the second moments of the inflows $\langle P_i P_j \rangle$. Given the network topology and the edge weights w_ℓ, we can further compute the moments of the edge flows $\langle F_\ell^2 \rangle$ using Kirchhoff's equations. Then one can proceed as in the original model as outlined above. Starting from an initial guess of the edge weights, we iterate the following two steps until convergence:

1. The moments of the flows $\langle F_\ell^2 \rangle$ are determined from the moments $\langle P_i P_j \rangle$ via Kirchhoff's equations.

2. The weights are updated according to Eq. (42).

Supplementary Note 5: Clustering in primal and dual graphs

While we used spectral clustering to find communities in both primal and dual graphs to enable an analytical treatment in the main manuscript, there are various different algorithms to determine the community structure in a given network. A commonly used and efficient algorithm is the *Louvain Community Detection Algorithm*, which is based on finding a community partition by iteratively optimizing the modularity of this partition. We are using the algorithm implemented in Networkx-2.8.6 [27], which uses a combination of [28] and [29].

The algorithm consists of two steps that are run iteratively. Starting from a partition in which every node is in its own community, the change in modularity ΔQ for removing an isolated node i from their lonely community and moving them to a neighboring community $\mathcal{C}$ is calculated using

$$\Delta Q = \frac{k_{i,\mathrm{in}}}{2m} - \xi\, \frac{\mathcal{S}_{\mathrm{tot}} \cdot k_i}{2m^2}$$

where m is the total number of edges in the graph, $k_{i,\mathrm{in}}$ is the sum of the weights of the edges connecting node i to the community $\mathcal{C}$, and $\mathcal{S}_{\mathrm{tot}}$ is the sum of the weights of all edges connected nodes in $\mathcal{C}$. A resolution parameter ξ smaller than 1 favors larger communities, while ξ larger than 1 favors smaller communities. The first step continues moving isolated nodes to neighboring communities until no further increase in modularity gain can be achieved. In the second step, the previously found communities are interpreted as nodes of a new network, and the weights of the new edges that connect the new nodes are given be the sum of the edges that connected the different communities. Subsequently, the first step can be executed again using this new network to find larger communities. These two steps are run iteratively until no increase in modularity that is larger than a small threshold is achieved.

Choosing a resolution parameter of $\xi = 0.01$ and running the clustering algorithm for the primal and dual graph of the *Acer platanoides* leaf, we find the community structure of the given graphs and compare them with the ones found by hierarchical spectral clustering. The results are shown in Fig. 6. Note, the set of edges that separate the communities are the edges connecting the different communities in the case of primal graphs, while they are along a path in the case of dual communities. In both cases the primal (i.e. original) graphs are shown in the background in each panel in Fig. 6. The separation along veins is even more pronounced and accurate when using the Louvain method, indicating that the description as dual graphs reveals important structural features of the topology of leaf venation networks.

SUPPLEMENTARY REFERENCES

[1] Corson, F. Fluctuations and Redundancy in Optimal Transport Networks. *Physical Review Letters* **104**, 048703 (2010).
[2] Newman, M. E. J. Modularity and community structure in networks. *Proceedings of the National Academy of Sciences* **103**, 8577–8582 (2006).
[3] Newman, M. E. J. *Networks: an introduction* (Oxford University Press, 2010).
[4] Miekkala, U. Graph properties for splitting with grounded Laplacian matrices. *BIT* **33**, 485–495 (1993).
[5] Diestel, R. *Graph Theory* (Springer, New York, 2000).
[6] Wood, A. J., Wollenberg, B. F. & Sheblé, G. B. *Power Generation, Operation and Control* (John Wiley & Sons, New York, 2014).
[7] Purchala, K., Meeus, L., Dommelen, D. V. & Belmans, R. Usefulness of dc power flow for active power flow analysis. In *IEEE Power Engineering Society General Meeting*, 454–459 Vol. 1 (2005).
[8] Van Hertem, D., Verboomen, J., Purchala, K., Belmans, R. & Kling, W. Usefulness of dc power flow for active power flow analysis with flow controlling devices. In *The 8th IEEE International Conference on AC and DC Power Transmission*, 58–62 (2006).
[9] Bollobás, B. *Modern graph theory*. No. 184 in Graduate texts in mathematics (Springer, 1998).
[10] Dörfler, F., Simpson-Porco, J. W. & Bullo, F. Electrical networks and algebraic graph theory: Models, properties, and applications. *Proceedings of the IEEE* **106**, 977–1005 (2018).
[11] Kirchhoff, G. Ueber die Auflösung der Gleichungen, auf welche man bei der Untersuchung der linearen Vertheilung galvanischer Ströme geführt wird. *Annalen der Physik* **148**, 497–508 (1847).
[12] Hwang, N. & Houghtalen, R. *Fundamentals of Hydraulic Engineering Systems* (Prentice Hall, Upper Saddle River, NJ, 1996).
[13] Díaz, S., González, J. & Mínguez, R. Observability analysis in water transport networks: Algebraic approach. *Journal of Water Resources Planning and Management* **142**, 04015071 (2016).
[14] Katifori, E., Szöllősi, G. J. & Magnasco, M. O. Damage and fluctuations induce loops in optimal transport networks. *Physical Review Letters* **104**, 048704 (2010).
[15] Moore, E. H. On the reciprocal of the general algebraic matrix. *Bull. Amer. Math. Soc.* **26**, 394–395 (1920).
[16] Ronellenfitsch, H., Manik, D., Hörsch, J., Brown, T. & Witthaut, D. Dual theory of transmission line outages. *IEEE Transactions on Power Systems* **32**, 4060 – 4068 (2017).
[17] Ronellenfitsch, H., Timme, M. & Witthaut, D. A dual method for computing power transfer distribution factors. *IEEE Transactions on Power Systems* **32**, 1007–1015 (2017).
[18] Strake, J., Kaiser, F., Basiri, F., Ronellenfitsch, H. & Witthaut, D. Non-local impact of link failures in linear flow networks. *New Journal of Physics* **21**, 053009 (2019).
[19] Kaiser, F., Latora, V. & Witthaut, D. Network isolators inhibit failure spreading in complex networks. *Nature Communications* **12**, 3143 (2021).
[20] Manik, D. *et al.* Network susceptibilities: Theory and applications. *Physical Review E* **95** (2017).
[21] Witthaut, D., Rohden, M., Zhang, X., Hallerberg, S. & Timme, M. Critical links and nonlocal rerouting in complex supply networks. *Physical Review Letters* **116**, 138701 (2016).
[22] Ballentine, L. E. *Quantum Mechanics – A Modern Development* (World Scientific, Singapore, 1998).
[23] Ben-Israel, A. & Greville, T. N. E. *Generalized inverses: theory and applications*. No. 15 in CMS books in mathematics (Springer, New York, 2003), 2nd ed edn.
[24] Kettemann, S. Delocalization of disturbances and the stability of ac electricity grids. *Physical Review E* **94** (2015).
[25] Labavic, D., Suciu, R., Meyer-Ortmanns, H. & Kettemann, S. Long-range response to transmission line disturbances in DC electricity grids. *The European Physical Journal Special Topics* **223**, 2517–2525 (2014).
[26] Jung, D. & Kettemann, S. Long-range response in ac electricity grids. *Physical Review E* **94**, 012307 (2016).
[27] Hagberg, A., Swart, P. & S Chult, D. Exploring network structure, dynamics, and function using networkx. Tech. Rep., Los Alamos National Lab.(LANL), Los Alamos, NM (United States) (2008).
[28] Traag, V. A., Waltman, L. & Van Eck, N. J. From louvain to leiden: guaranteeing well-connected communities. *Scientific reports* **9**, 1–12 (2019).
[29] Blondel, V. D., Guillaume, J.-L., Lambiotte, R. & Lefebvre, E. Fast unfolding of communities in large networks. *Journal of statistical mechanics: theory and experiment* **2008**, P10008 (2008).
